# Human 3D Ovarian Cancer Models Reveal Malignant Cell–Intrinsic and –Extrinsic Factors That Influence CAR T-cell Activity

**DOI:** 10.1158/0008-5472.CAN-23-3007

**Published:** 2024-05-31

**Authors:** Joash D. Joy, Beatrice Malacrida, Florian Laforêts, Panoraia Kotantaki, Eleni Maniati, Ranjit Manchanda, Alessandro Annibaldi, Sarah Hopkins, Ianire Garrobo-Calleja, Julien Gautrot, Frances R. Balkwill

**Affiliations:** 1 Barts Cancer Institute, Queen Mary University of London, London, United Kingdom.; 2 Wolfson Institute of Population Health, Cancer Research UK, Barts Centre, Queen Mary University of London, London, United Kingdom.; 3 Department of Gynaecological Oncology, Royal London Hospital, Barts Health NHS Trust, London, United Kingdom.; 4 Department of Health Services Research and Policy, London School of Hygiene and Tropical Medicine, London, United Kingdom.; 5 Centre for Molecular Medicine Cologne, University of Cologne, Cologne, Germany.; 6 GlaxoSmithKline Medicines Research Centre, Stevenage, United Kingdom.; 7 School of Engineering and Material Science, Centre for Bioengineering, Queen Mary University of London, London, United Kingdom.

## Abstract

*In vitro* preclinical testing of chimeric antigen receptor (CAR) T cells is mostly carried out in monolayer cell cultures. However, alternative strategies are needed to take into account the complexity and the effects of the tumor microenvironment. Here, we describe the modulation of CAR T-cell activity by malignant cells and fibroblasts in human three-dimensional (3D) *in vitro* cell models of increasing complexity. In models combining mucin-1 (MUC1) and TnMUC1 CAR T cells with human high-grade serous ovarian cancer cell spheroids, malignant cell–intrinsic resistance to CAR T-cell killing was due to defective death receptor signaling involving TNFα. Adding primary human fibroblasts to spheroids unexpectedly increased the ability of CAR T cells to kill resistant malignant cells as CCL2 produced by fibroblasts activated CCR2/4^+^ CAR T cells. However, culturing malignant cells and fibroblasts in collagen gels engendered production of a dense extracellular matrix that impeded CAR T-cell activity in a TGFβ-dependent manner. A vascularized microfluidic device was developed that allowed CAR T cells to flow through the vessels and penetrate the gels in a more physiological way, killing malignant cells in a TNFα-dependent manner. Complex 3D human cell models may provide an efficient way of screening multiple cytotoxic human immune cell constructs while also enabling evaluation of mechanisms of resistance involving cell–cell and cell–matrix interactions, thus accelerating preclinical research on cytotoxic immune cell therapies in solid tumors.

**Significance:**

Three-dimensional *in vitro* models of increasing complexity uncover mechanisms of resistance to CAR T cells in solid tumors, which could help accelerate development of improved CAR T-cell constructs.

## Introduction

The aim of this paper is to investigate the potential of human three-dimensional (3D) cancer cell models in understanding barriers to chimeric antigen receptor (CAR) T-cell therapy in solid tumors. We have focused on models of high-grade serous ovarian cancer (HGSOC), a disease with poor response to immunotherapy (1, 2).

A better understanding of immune regulation in tumor microenvironment (TME) has led to rapid development of immunotherapies that “re-program” host immune cells to induce antitumor effects, but activity varies with tumor type and stage (3). One successful approach is CAR T-cell therapy involving genetically engineering T cells to express receptors that can recognize cell surface tumor antigens, “re-directing” the cytotoxic potential of T cells against malignant cells (4). CAR T-cell therapy has led to impressive clinical responses in some hematological cancers (5, 6) but has limited efficacy in solid tumors (7, 8). The modest response to CAR T-cell therapy in solid tumors might be due to the complex immunosuppressive microenvironment, which includes anti-inflammatory cytokines, immunosuppressive cells, immune checkpoints, and abnormal extracellular matrix (ECM) that collectively deter interactions of immune cells and malignant cells (4, 9). Therefore, we need to understand the dynamic interaction between CAR T cells and the TME to elucidate resistance of CAR T-cell therapy in solid tumors and improve clinical efficacy.

Human malignant cell monocultures and mouse cancer models are commonly used to test novel therapies such as CAR T cells. However, two-dimensional (2D) cultures do not recapitulate the complex and dynamic TME (10). Murine models are often difficult, labor intense, and expensive to run at large scale (11). Mouse models might not be ideal for understanding the human TME as they may lack well-developed TMEs that are found in the advanced disease of most clinical trial patients. Furthermore, several immunotherapies such as CAR T cells utilize humanized antibodies and mouse phenocopies may not accurately reflect their mechanisms of action (12). Fifty-four percent of novel therapeutics fails in clinical trials due to inadequate efficacy and/or safety concerns (13). More efficient preclinical screening tools are required to identify ideal drug candidates for *in vivo* testing, thus preventing poor candidates from entering clinical trials.

Three-dimensional *in vitro* human cell models could address these issues. These models recapitulate some of the biomechanical and biochemical cues that are vital for tumor progression (14). Apart from medium to high throughput and reproducibility, 3D *in vitro* models offer the power to deconvolute the TME to study specific cell–cell and cell–matrix interactions, which is not always possible with preclinical animal models (15). Microfluidic cell culture is a novel technology, which utilizes micro-engineered chips with perfusable channels seeded with cells to replicate some elements of *in vivo* tissue architecture and function (16). Physiologically relevant mechanical cues such as fluid flow through microvasculature can be achieved within microfluidic *in vitro* cell models (16). Recently, the US Food and Drug Administration (FDA)-approved alternatives to animal models, such as physiologically relevant human cell models for drug safety and efficacy testing (17).

Here we describe how we have used human HGSOC cell models with increasing complexity to investigate barriers to CAR T-cell activity. We have identified malignant cell–intrinsic resistance to CAR T-cell killing and uncovered mechanisms by which fibroblasts may positively and negatively influence CAR T-cell activity. Finally, we report the use of vascularized microfluidic ovarian cancer models to investigate CAR T-cell migration and cytotoxicity. We conclude that building human cell models of increasing complexity may accelerate preclinical research into CAR T-cell and other immune cell constructs in solid tumors.

## Materials and Methods

### Cell culture

G164 and G33 cells were established in our laboratory from omental HGSOC tumors. Both cells had p53 mutation and were positive for PAX8. G164 cells were cultured in DMEM/F12 (Gibco, Cat. 31331093) with 4% human serum (Sigma, Cat. H4522) and 1% penicillin-streptomycin (pen/strep; Sigma, Cat. P4333). G33 cells were cultured in DMEM/F12 with 10% fetal bovine serum (FBS) and 1% pen/strep. HGSOC cell line OvCAR3 (ATCC, Cat. HTB-161) and ductal carcinoma cell line T47D (ATCC, Cat. HTB-133, RRID: CVCL_0553) were cultured in RPMI 1640 medium (Gibco, Cat. 21875034) supplemented with 10% FBS and 1% pen/strep. NALM6 (ATCC, Cat. CRL-3273, RRID: CVCL_4V57) cells were cultured in RPMI 1640 medium with 20% FBS and 1% pen/strep. Human umbilical vein endothelial cells (HUVEC; Lonza, Cat. C-2519A, RRID: CVCL_2959) and red fluorescent protein (RFP)-labeled HUVEC (Angio-Proteomie, Cat. cAP-0001RFP) were cultured in endothelial growth medium 2 (EGM2; Promocell, Cat. C22011). Quality control of cell lines was carried out by frequent STR analysis (ATCC), *Mycoplasma* testing (InvivoGen), and all cells were used within four to five passages after thawing.

### Patient samples and study approval

Fresh macroscopically normal omental tissues were kindly donated by patients with gynecological cancer undergoing surgery at Barts NHS Trust, London. Omental tissues deemed by a lead surgeon to be surplus to diagnostic and therapeutic requirement were collected with written informed consent from patients. All tissues used for this study was approved by a UK review board (REC 17/LO/0405). Experiments were conducted in accordance with the Declaration of Helsinki and International Ethical Guidelines for Biomedical Research involving Human Subjects.

### Omental fibroblast isolation

Omental fibroblasts were isolated as described by previously (18). Briefly, omental samples were diced into small pieces and were digested using 1 mg/mL liberase TL (Sigma, Cat. 5401020001). The digested tissue was filtered through 250 μm tissue strainers (Thermo Fisher Scientific, Cat. 87791). Filtered tissue digest was centrifuged and the cell pellet, which composed of stromal vascular fraction was resuspended in DMEM/F12 with 10% FBS and 1% pen/step and plated in T75 flasks. Cells were cultured until the flasks were confluent. Omental fibroblasts were then either frozen down or used for subsequent experiments between passages 2 to 4. Fibroblasts were previously characterized using different markers such as FAP and αSMA (19).

### Collagen gel

Collagen gel solution (1 mg/mL) was made by mixing (for 100 μL gel) 33 μL of 3 mg/mL rat-tail collagen (Thermo Fisher Scientific, Cat. A1048301), 10 μL of 10× DMEM low glucose (Sigma, Cat. D2429), 1.7 μL of 1 mol/L sodium hydroxide (NaOH; Sigma-Aldrich, Cat. 221465), 5.3 μL of sterile-filtered water (Sigma-Aldrich, Cat. W3500), and 50 μL of cells suspended in complete medium. All preparation was carried out on ice to prevent premature gelation. A total of 100 μL of cells suspended in collagen solution were pipetted into a 96-well plate and incubated in a 37°C for 30 minutes to allow gelation. Gels were carefully scooped out and transferred to a 24-well plate containing culture medium. Medium was replaced every 2 to 3 days.

Monoculture gels were seeded with 100k cells and coculture were seeded at a ratio of 1:1 (50k:50k) fibroblasts to malignant cells. For triculture gels, cells were seeded at 2:2:1 (40k:40k:20k) ratios of fibroblasts to malignant cells to HUVECs.

### Lentivirus production

PEIpro transfection reagent (Polyplus, Cat. 115-100) was used to transfect 2 × 10^6^ suspension adapted HEK293T cells/mL with 0.15 μg/mL pKL(gag.pol), 0.025 μg/mL pKR(rev), 0.075 μg/mL pKG(VSVg), and 0.5 μg/mL transfer plasmid containing CD19, MUC1, and TnMUC1 single-chain variable fragments. Twenty-four hours post-transfection, 5 mmol/L of sodium butyrate (Sigma, Cat. 303410) was added and cells were cultured at 37°C with 5% CO_2_ at 110 rpm. Three days post-transfection, cell supernatant was filtered through 0.8 μm (Thermo Fisher Scientific, Cat. 450-0080) and 0.45 μm Nalgene filters (Thermo Fisher Scientific, Cat. 450-0045) and then treated with 50 U/mL benzonase (Sigma, Cat. E1014) at 37°C for 30 minutes. Supernatant was ultracentrifuged at 70,000 × *g* for 2 hours at 4°C with 20% sucrose cushion. Lentivirus pallets were resuspended in buffer containing 20 mmol/L tromethamine (Sigma-Aldrich, Cat. T6687), 100 mmol/L sodium chloride (Sigma-Aldrich, Cat. 71380), 10 mg/mL sucrose (Thermo Fisher Scientific, Cat. BPE220-1), and 10 mg/mL D-mannitol (Sigma-Aldrich, Cat. M9546) and were stored at −80°C.

### CAR T-cell generation

Blood (Research Donors) from healthy donors was centrifuged in ACCUSPIN tubes (Sigma-Aldrich, Cat. A2055) with Histopaque-1077 (Sigma-Aldrich, Cat. 10771) at 800 × *g* for 15 minutes with no deceleration to isolate peripheral blood mononuclear cells (PBMC). The human biological samples were sourced ethically and their research use was in accord with the terms of the informed consents under an IRB/EC approved protocol. PBMCs were treated with 1× red blood cell lysis solution (BioLegend, Cat. 420301) and then cultured in TexMACS medium (Miltenyi Biotec, Cat. 130-097-196) with 1 mg/mL of IL2 (Sigma, Cat. SRP3085) and 1:100 TransAct T-cell activation beads (Miltenyi Biotec, Cat. 130-111-160). Two days after PBMC isolation, 1 × 10^6^ T cells were transduced with lentiviruses at multiplicity of infection of 5 and centrifuged at 800 × *g* for 2 hours at 37°C. Transduced T cells were transferred to G-Rex plates (Wilson Wolf, Cat. 80192M) 2 days post-transduction and were cultured for 10 days with medium changed every 2 to 3 days. Twelve days post-transduction, T cells were frozen down in CryStor CS5 freezing medium (Sigma-Aldrich, Cat. C2999).

### Treating target cells with CAR T cells

For monolayer cultures, 20,000 malignant cells were seeded in 96-well plates. Malignant cells were cocultured with primary fibroblasts at 1:1 (10k:10k) ratio. Cells were cultured in T-cell assay medium [Phenol red–free RPMI 160 medium (Thermo Fisher Scientific, Cat. 11835063) with 10% FBS and 1% pen/strep]. Cells were cultured overnight to let the cell adhere before CAR T-cell treatment.

For suspension spheroid cultures, 2,000 malignant cells were seeded in 96-well, round bottom, ultra-low attachment plates (Sigma-Aldrich, Cat. CLS7007). Malignant cells were cocultured with fibroblasts at 1:1 (1k:1k) ratio. Cells were cultured in T-cell assay medium. Plates were centrifuged at 300 × *g* for 5 minutes and incubated overnight prior to CAR T-cell addition.

Coculture collagen gels were made as described above. G164/fibroblast gels were cultured with or without 20 μmol/L of TGFβ receptor inhibitor SB431542 (Sigma-Aldrich, Cat. S417). Gels were cultured for 14 days and then transferred to T-cell assay medium prior to CAR T-cell treatment.

Thawed CAR T cells were resuspended in T-cell assay medium and were added to the target cells at 1:1, 1:5, or 1:10 target-to-effector ratio. The ratio of effector cells refers to the ratio of T cells that were successfully transduced to produce CAR T cells. For malignant cell and fibroblast coculture experiments, the ratio of target cells refers to the number of malignant cells. For collagen gel experiments, the ratio of target cells refers to number of malignant cells at the day of gel casting. Target cells were treated with CAR T cells for 2 to 3 days. Target cell and CAR T-cell cultures were treated with 0 to 25 μg/mL of pembrolizumab (Merck), 1 μmol/L of birinapant TL32711 (Selleckchem, Cat. S7015), 5 ng/mL recombinant CCL2 (R&D Systems, Cat. 279-MC-010), 5 μg/mL anti-CCL2 antibody (R&D Systems, Cat. MAB679), or 5 μg/mL anti-TNFα antibody (Thermo Fisher Scientific, Cat. AMC3012).

### Enzyme-linked immunosorbent assay

Enzyme-linked immunosorbent assay (ELISA) was performed using human IFNα Quantikine kit (R&D Systems, Cat. DIF50C), human TNFα QuantiGlo kit (R&D Systems, Cat. QTA00C), human CCL2/MCP1 Quantikine kit (R&D Systems, Cat. DCP00), and human TGFβ1 Quantikine kit (R&D Systems, Cat. DB100C) according to manufacturer’s instructions. ELISA plates were read immediately using FLUOstar optima microplate reader (BMG Labtech).

### Mesoscale discovery

Mesoscale discovery (MSD) was performed using human IFNγ tissue culture kit (MSD, Cat. K151AEB-2) and human pro-inflammatory panel 1 kit (MSD, Cat. K15049D-1) according to manufacturer’s instructions. Plates were read immediately on MSD sector 600 imager (MSD).

### siRNA transfection

A total of 250,000 primary omental fibroblasts were resuspended in Opti-MEM medium (Thermo Fisher Scientific, Cat. 51985034) and plated in a six-well plate. CCL2 siRNA (0.8 mmol/L; Qiagen, Cat. 1027416) and Lipofectamine 3000 (Thermo Fisher Scientific, Cat. L3000015) were added while the cells were still in suspension. Twenty-four hours after transfection, Opti-MEM medium was replaced with fibroblast culture medium and cells were then cultured for 2 days.

### Incucyte cytotoxicity assay

Cocultures of CAR T cells with target cells in monolayer and suspension spheroids were set up as described above. For monolayer cultures, 96-well plates were coated with poly-L-ornithine (Sigma-Aldrich, Cat. P4957) overnight at 4°C. Plates were thoroughly washed with sterile water prior to seeding target cells. Cells were cultured with Incucyte Cytotox Red Reagent (Sartorius, Cat. 4632) diluted in T-cell assay medium at 1:4,000 for 1 hour at 37°C prior to the addition of CAR T cells. After CAR T cells were added, the plates were placed in the humidified Incucyte S3 (Essen Bioscience) at 37°C with 5% CO_2_. Images were acquired every 2 hours for 3 days. The software Incucyte S3 v2018C (Essen Bioscience) was used to analyze the images by measuring the change in total red area that depicted cell death. The total red area was normalized to obtain percentage live cells by using the following equations:

Coculutre background response (CBG)=Coculture red area-(effector only+target only red area) (A)

$$\%\;\text{Live}\;\text{cells}=\frac{\text{Maximum}\;\text{CBG}-{\text{CBG}}^{t=n}}{\text{Maximum}\;\text{CBG}-{\text{CBG}}^{t=1}}\times 100$$ (B)

CBG^*t*=*n*^ refers to CBG at a given time point and CBG^t=1^ refers to CBG at the first time point.

### Western blot

Cells were lysed with RIPA buffer (Sigma, Cat. R0278) containing 1:10 protease inhibitors (Roche, Cat. 11836153001) and 1:100 phosphatase inhibitors (Sigma, Cat. P0044). Protein concentration was determined using BCA assay. A total of 25 μg of the sample was loaded with 4% to 12% NuPAGE Bis-Tris gels (Invitrogen, Cat. NP0321BOX). Samples were run in 1× NuPAGE running buffer (Invitrogen, Cat. NP0001) and transferred onto a membrane (Immobilon, Cat. IPVH00010) in 1× NuPAGE transfer buffer (Invitrogen, NP0006-1). The membrane was blocked in 5% bovine serum albumin (BSA; Sigma, Cat. A8022) for 1 hour at room temperature. The membrane was incubated with cIAP2 antibody (R&D Systems, Cat. AF8171) at 4°C overnight, followed by incubation with anti-goat horseradish peroxide (HRP)-conjugated antibody (Dako, Cat. P0160) for 1 hour at room temperature. HRP activity was visualized with Amersham Imager 600 (GE Healthcare).

### Proteome profiler array

Proteome profiler array was performed using human cytokine array kit (R&D Systems, Cat. ARY005B) according to manufacturer’s instructions. The membrane was imaged on Amersham Imager 600 (GE Healthcare).

### RNA isolation and sequencing

RNA isolation was performed using RNeasy microkit (Qiagen, Cat. 74004) according to manufacturer’s instructions. RNA sequencing was performed by the Wellcome Trust Centre for Human Genetic to an average of 67 million reads per sample. Library preparation was performed using ribosomal depletion and a strand-specific protocol and sequencing was carried out generating 150 base pairs (bp) paired end reads. FastQC v0.11.5 was performed and quality trimming was applied with seqtk v1.2-r94. Alignment to the reference genome GRCh38 was performed using STAR v2.7.0f alignment with two-pass procedure that increases sensitivity to novel splice junctions (PMID: 26334920). Counting of the reads was performed with RSEM v1.3.1 using the ensemble annotation GRCh38.101. Only genes that achieved at least one transcript per million in at least 25% of the samples were kept and a log_2_ transcript per million–normalized gene expression matrix was generated. Differential expression analysis was performed using the lm model of the limma R package and voom normalization with a ∼0+group model design. Gene set enrichment analysis (GSEA) was performed on GenePattern v7.2.4 using ranked t-statistic of all genes for canonical pathways (c2.cp.v7.4.symbols.gmt). RNA sequencing FASTQ files are available on the Gene Expression Omnibus (GEO) database under the accession number GSE235099.

### Flow cytometry

Collagen gels were digested using 1 mg/mL of collagenase type I (Thermo Fisher Scientific, Cat. 17100017). Cells were stained with 1:500 anti-MUC1 HMFG2 (Absolute antibody, Cat. Ab00715-1.1), 1:400 anti-TnMUC1 5E5 (Creative Biolabs, Cat. TAB-418MZ), 1:500 F(ab′)_2_ (Jackson immunoresearch, Cat. 115-066-072), and 1:200 ProteinL (Thermo Fisher Scientific, Cat. 29997) diluted in fluorescence-activated cell sorting (FACS) buffer—phosphate-buffered saline (PBS; Sigma-Aldrich, Cat. D8537) with 2 mmol/L EDTA (Thermo Fisher Scientific, Cat. AM9262) and 2.5% BSA. Cells were then incubated with 1:250 anti-mouse AF488 (Thermo Fisher Scientific, Cat. A-11029) and 1:1,000 streptavidin-APC (Biolegend, Cat. 405243). Subsequently, cells were incubated with fluorophore-conjugated antibodies (Supplementary Table S1) and then with 1:1,000 fixable viability dye eFluor 450 (Thermo Fisher Scientific, Cat. 65-0863-14), eFluor 506 (Thermo Fisher Scientific, 65-0866-18), or SYTOX red (Thermo Fisher Scientific, Cat. S34859). Cells were fixed in 2% formalin, resuspended in 200 μL of FACS buffer, and stored at 4°C. Data were acquired on LSR fortessa cell analyzer (BD Biosciences) and analyzed on FlowJo v10.1.5 (BD Biosciences).

### Immunohistochemistry

Collagen gels were fixed in formalin (Sigma, Cat. HT501128) overnight at 37°C and then embedded in 2% agarose (Thermo Fisher Scientific, Cat. 10264544) prior to paraffin embedding and sectioning (4 μm). Human tissues were fixed in formalin, dehydrated in 70% ethanol, and then embedded in paraffin for sectioning. Sections were deparaffinized in two changes of xylene and rehydrated through 100%, 90%, 70%, and 50% ethanol solutions. No antigen unmasking was required for fibronectin staining. For collagen 1 alpha 1 (COL1A1) staining, antigen unmasking was done using 1 mg/mL collagenase IV (Gibco, Cat. 17104-019) in 1× Hanks’ Balanced Salt Solution (Gibco, Cat. 14185-045) supplemented with 0.001 mol/L calcium chlorine (Sigma-Aldrich, Cat. C8106) at room temperature for 1 hour. For CD3 and CD8 staining, 1× antigen retrieval Tris-EDTA buffer, pH 9.0 (Abcam, Cat. ab93684) was used. For all the other staining, 1× citric acid–based antigen retrieval buffer (Vector Laboratories, Cat. H-3300) was used. Slides were placed in preheated antigen retrieval buffer for 30 minutes in 95°C water bath. Peroxidase activity was blocked using 0.6% hydrogen peroxide (H_2_O_2_; Thermo Fisher Scientific, Cat. H/1800/15) in methanol for PAX8, Ki67, CD3, caspase-3 staining, and 3% H_2_O_2_ in PBS for other staining. All sections were then further blocked with 2.5% goat serum (Life Technologies, Cat. 16210064) + 2.5% BSA and subsequently incubated with primary antibody (Supplementary Table S2) overnight. Detection was performed with Impress goat anti-rabbit (Vector Laboratories, Cat. MP-7451-50) or anti-mouse (Vector Laboratories, Cat. MP-7452-15) IgG polymer kit, followed by incubation with DAB chromogen (Dako, Cat. K346811-2). Sections were counterstained in Gill’s I hematoxylin (Merck, Cat. CI75290), dehydrated through 50%, 70%, 90%, and 100% ethanol, followed by two changes in xylene. Sections were mounted in DPX resin (Sigma-Aldrich, Cat. 06522) and imaged the day after using a Pannoramic 250 high throughput scanner (3DHistech) or NanoZoomer S210 slide scanner (Hamamatsu) and the scans were analyzed using Definiens.

### Masson’s trichrome

Paraffin-embedded sections were deparaffinized and rehydrated as described above. Sections were then incubated in Bouin’s solution (Sigma-Aldrich, Cat. HT10132) overnight and subsequently incubated in 1:1 ratio of Weigert’s A and Weigert’s B hematoxylin solution (Sigma-Aldrich, Cat. HT1079). Masson’s trichrome staining was performed using the Masson’s trichrome staining kit (Sigma-Aldrich, Cat. HT15). Sections were incubated in Biebrich scarlet-acid fuchsin solution, followed by phosphotungstic/phosphomolybdic acid solution and aniline blue solution and finally 1% acetic acid (Sigma-Aldrich, Cat. 1000631011). Slides were dehydrated very quickly in two changes of 90% and 100% ethanol, followed by two changes in xylene, mounted in DPX resin overnight, and imaged using Pannoramic 250 high throughput scanner (3DHistech) or NanoZoomer S210 slide scanner (Hamamatsu).

### ECM density characterization

High-density matrix (HDM) of ECM was characterized as described previously (20). Briefly, digital images from collagen gel sections were obtained using the NPD.view 2.7.25 software under 20× magnification. FIJI/ImageJ (v.1.53m) image processing and analysis program (NIH) was used to color deconvolute, selecting DAB filter for fibronectin and COL1A1 staining and H PAS filter for Masson’s trichrome. Deconvoluted images were analyzed for TWOMBLI (the workflow of matrix biology informatics) using TWOMBLI v1 plugin. Two representative images from each staining were used as a test set to determine optimal parameters for contrast saturation and maximum display HDM. The mean of the TWOMBLI metrics was calculated per gel and displayed on the graphs.

### Collagen gel slicing, imaging, and T-cell behavior analysis

Collagen gel slicing, imaging, and T-cell behavior analysis were done as described previously (21). Briefly, collagen gels were embedded in 5% low gelling temperature agarose (Merck, Cat. A0701) and sliced with Leica VT1200S Vibratome with slice thickness of 350 μm. Slices were transferred to a petri dish and a stainless-steel ring was placed around the collagen gel to hold AF405-conjugated fibronectin (Novus biologicals, Cat. NBP2-34633) and AF594-conjugated EpCAM (BioLegend, Cat. 324202) antibodies diluted 1:10 in phenol red–free RPMI 1640 medium for 15 minutes at 37°C. Slices were then transferred to a glass bottom dish, covered with phenol red–free RPMI 1640 medium and secured with a tissue slice anchor.

Collagen gel slices were imaged using Nikon TE Eclipse confocal microscope equipped with a spinning disk and a temperature-controlled chamber set at 37°C, with 5% CO_2_. Phenol red–free RPMI 1640 medium bubbled with 95% O_2_ and 5% CO_2_ was perfused into the imaging dish at 1 mL/minutes. Slices were imaged every 30 seconds for 30 minutes. Images were analyzed using Imaris v9.1 (Oxford Instruments) and migration metrics were aggregated in R v4.2.2 and RStudio 2022.12.0.353, using a custom built R script, to categorize T cells as static (maximum displacement < 7 μm and trajectory length < 13 μm), wobbling (maximum displacement < 7 μm and trajectory length ≥ 13 μm), migrating (maximum displacement 7–25 μm and trajectory length 13–40 μm), and long migrating (maximum displacement > 25 μm and trajectory length > 40 μm).

### Fabrication of microfluidic device

A silicon wafer (PI-KEM) with positive relief pattern of SU-8 2050 (A-gas electronic materials) was produced by photolithography. This master was used to obtain negative replicas by casting polydimethylsiloxane (PDMS; Ells-worth adhesives) against the master and curing at 60°C overnight. Upon separating from the master, gel ports, the central well, and medium reservoirs were punched into the PDMS chips using biopsy punches. The PDMS chips were then bonded onto glass coverslips using an oxygen plasma treatment. The devices were autoclaved and placed in a 70°C oven for at least 3 days to restore hydrophobicity.

### Cell culture in microfluidic device

HUVECs resuspended in thrombin solution (Sigma-Aldrich, Cat. T6634) were mixed with bovine fibrinogen (Sigma-Aldrich, Cat. F8630) to achieve final cell density, fibrinogen, and thrombin concentrations of 6 × 10^6^ cells/mL, 10 mg/mL, and 2 U/mL, respectively. Fibrin gel precursor solutions were slowly injected into the central channel through the gel loading ports and allowed to cross-link at 37°C for 5 minutes, after which, EGM2 medium was loaded to the inlets of the two side channels and suction was applied at the corresponding outlets to fill the hydrophobic medium channels.

Once fibrin gels were polymerized, collagen gels were placed on top of the central well and were pushed down gently using a p10 pipette tip. The wells were then further supplemented with fibrin gel. Medium reservoirs were filled with EGM2 medium supplemented with 50 ng/mL vascular endothelial growth factor (VEGF; PeproTech, Cat. 100-20A). Devices were cultured for 7 days in a humidified incubator at 37°C and 5% CO_2_ with medium changed every 24 hours.

A total of 500,000 CAR T cells were resuspended in 360 μL EGM2 medium supplemented with 50 ng/mL VEGF. Suspensions of 100, 90, 90, and 80 μL of CAR T cells were added to top-left, bottom-left, top-right, and bottom-right medium reservoirs, respectively. The volume of medium was varied to create a pressure difference, thus creating a flow through the device (22). The device was cultured for 3 days with medium changed every 24 hours by carefully removing only 30 μL of medium from the top of the four medium reservoirs and replenishing 50, 40, 40, and 30 μL of fresh medium in top-left, bottom-left, top-right, and bottom-right medium reservoirs, respectively.

### Immunofluorescence

Medium from the four reservoirs of the microfluidic chip was replaced with PBS to wash the device 3 times for 5 minutes. Samples were then fixed with formalin for 15 minutes, permeabilized for 15 minutes using 0.2% Triton-X (Sigma-Aldrich, Cat. T8787), and blocked in 5% BSA for 4 hours at room temperature. Subsequently, chips were incubated with 1:100 dilution of AF647 anti-human CD31 antibody (Biolegend, Cat. 303112) for overnight at 4°C and then chips were incubated with DAPI diluted in PBS (1:1,000) for 30 minutes at room temperature. The devices were then covered in PBS and stored at 4°C, protected from light until imaged using Zeiss LSM 710 (Zeiss) at 10× objectives.

### Statistical analysis

Graphic representation of data and statistical analysis were performed using Prism v9.4.1 (GraphPad). Kolmogorov–Smirnov test was used to determine whether the data was normally distributed. Data that were normally distributed were subjected to two-sided student *t* test for the analysis of differences between two groups. Mann–Whitney test was used for nonparametric data. One- or two-way analysis of variance (ANOVA) was used for the assessment of difference between three or more groups. A *P* value of less than 0.05 was considered as statistically significant.

### Data availability

RNA sequencing FASTQ files are available on the GEO database under the accession number GSE235099. All data are available in the main text or the Supplementary Materials. Data generated and/or analyzed in the current study are available from the corresponding author upon reasonable request.

## Results

### OvCAR3 cells were sensitive but G164 cells were resistant to CAR T-cell cytotoxicity in monolayer cultures

**Figure 1.**
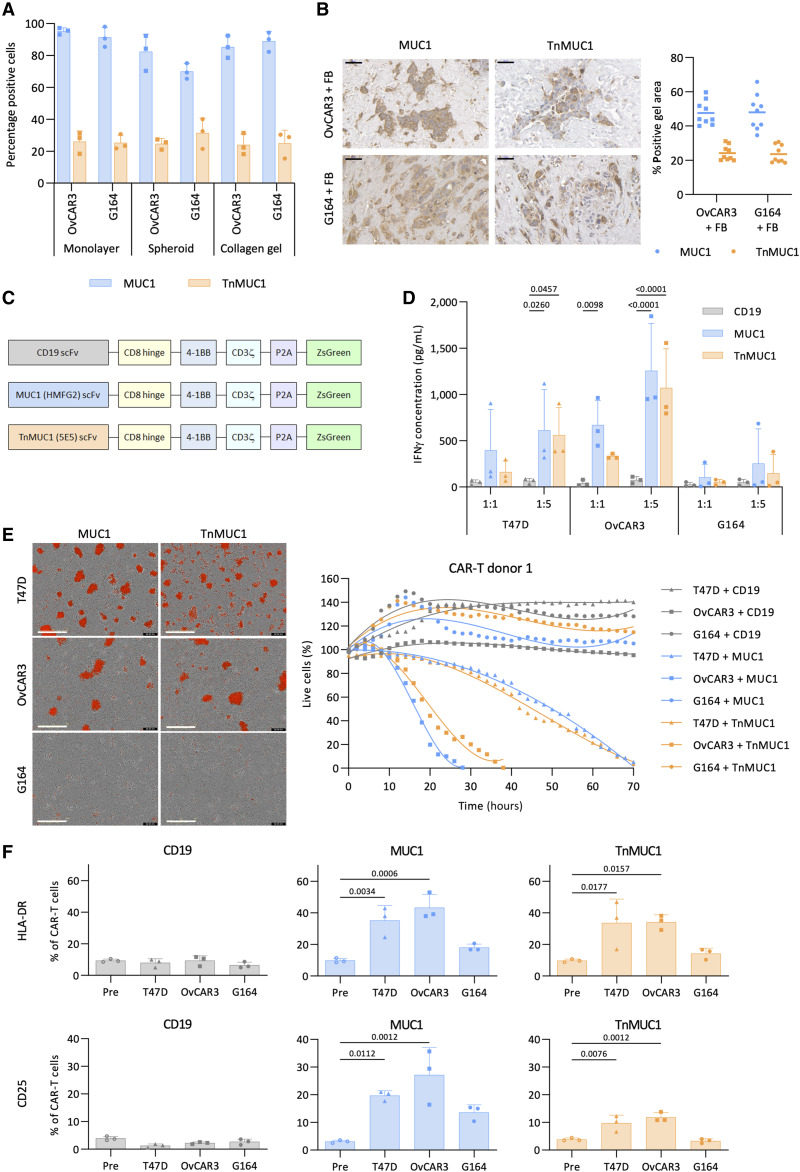
OvCAR3 cells were sensitive but G164 cells were resistant to CAR T-cell cytotoxicity in monolayer cultures. **A,** Flow cytometry analysis of the expression of MUC1 and TnMUC1 on monolayer, spheroid, and collagen gel cultures of OvCAR3 and G164 cells. Data plotted as mean ± SD of three repeats. **B,** Expression of MUC1 and TnMUC1 on OvCAR3+FB and G164+FB collagen gels. Data plotted as mean ± SD of three gels per three replicates. Different FB donors were used for each replicate. Scale bars, 50 μm. **C,** Schematic representation of CD19, MUC1, and TnMUC1 directed second generation CARs. **D,** ELISA data showing IFNγ concentration after coculturing CAR T cells with monolayers of T47D, OvCAR3, and G164 cells for 2 days at 1:1 and 1:5 T:E ratios. **E,** Representative images (left) and quantification (right) of Incucyte killing assay in which monolayers of T47D, OvCAR3, and G164 cells were treated with CAR T cells from one donor at 1:5 T:E ratio. Images shown are 3 days after treatment. Red, dead cells. Scale bars, 400 μm. **F,** Flow cytometry analysis of the expression of T-cell activation markers HLA-DR (top) and CD25 (bottom) pre and 2 days post coculture with monolayers of T47D, OvCAR3, and G164 cells. **D** and **F,** Data plotted as mean ± SD for three CAR T-cell donors. Statistics performed using two-way (**D**) and one-way ANOVA (**F**).

To assess CAR T-cell activity in models of HGSOC tumors, we first identified suitable target antigens that were expressed by HGSOC cell lines in monolayer, spheroids, and collagen gels. Mucin 1 (MUC1) is a transmembrane glycoprotein, which is highly glycosylated in normal cells but hypoglycosylated in malignant cells (23). TnMUC1 is an aberrant glycoform of MUC1 where the glycosylation terminates with the addition of N-acetylgalactosamine to the protein backbone (23). Two HGSOC cell lines, OvCAR3 and G164 (24) had comparable levels of MUC1 and TnMUC1 expression in monolayer, spheroids, and collagen gels (Fig. 1A). Both cell lines maintained MUC1 and TnMUC1 expression when cocultured with primary human omental fibroblasts (FB) from three different donors in collagen gels for 14 days (Fig. 1B). We also confirmed that MUC1 and TnMUC1 were highly expressed in human HGSOC omental tumors but there was low expression in adjacent omentum (Supplementary Fig. S1A) as reported by others in omentum and other sites of HGSOC (25).

Using T cells from three healthy donors, we then engineered second generation CAR T cells with 4-1BB costimulatory domain and ZsGreen fluorescent tag (Fig. 1C). The single-chain variable fragments of MUC1 and TnMUC1 CARs were derived from HMFG2 and 5E5 antibodies, respectively, both binds to hypoglycosylated MUC1. We also generated CAR T cells against the B-cell surface antigen CD19 as a negative control. CAR T-cell transduction efficiency was assessed using ZsGreen and cell surface CAR expression (Supplementary Fig. S1B). CAR T cells from all three donors had a transduction efficiency of approximately 40%. The ratio of CD8^+^/CD4^+^ CAR T cells was almost 1:1 and majority of the CAR T cells had CD45RA^−^ CCR7^+^ central memory phenotype (Supplementary Fig. S1C).

Our next step was to test the CAR T-cell activity in monolayer cultures of OvCAR3 and G164 cells. We used T47D, a ductal carcinoma cell line, as MUC1 and TnMUC1 positive control (Supplementary Fig. S1D). We assessed CAR T-cell activation by measuring interferon (IFNγ) concentration after coculturing CAR T cells with malignant cell monolayers for 2 days at different target-to-effector (T:E) ratios (Fig. 1D). As expected, CD19 CAR T cells showed no evidence of activation, but both MUC1 and TnMUC1 CAR T cells were significantly activated when cocultured with T47D and OvCAR3 cells at 1:5 T:E ratio. However, unexpectedly, IFNγ production was low when CAR T cells were cocultured with G164 cells even though they expressed both antigens. As measured in the Incucyte platform, MUC1 and TnMUC1 CAR T cells were cytotoxic against T47D and OvCAR3 cells but had no activity on G164 cells during 3 days of coculture (Fig. 1E). These results were replicated with CAR T cells from two different donors (Supplementary Fig. S1E). TnMUC1 CAR T cells exhibited highly effective killing despite only a proportion of target cells expressed the antigen. This might be because IFNγ produced by activated CAR T cells further induced the expression of TnMUC1 on the target cell, however, the mechanism for this is not fully known (26). CD19 CAR T cells were cytotoxic against NALM6, a CD19 positive B-cell precursor leukemia cell line (Supplementary Fig. S1F).

We next tried a lower T:E ratio (Supplementary Fig. S1G) and also treated G164 and CAR T-cell cocultures with pembrolizumab to see if immune checkpoint expression was inhibiting CAR T-cell activity (Supplementary Fig. S1H) but neither condition improved CAR T-cell activation against G164 cells. However, MUC1 and TnMUC1 CAR T cells cocultured with G164 cells produced higher levels of IFNγ compared to CD19 CAR T cells (Supplementary Fig. S1G and S1H), suggesting that G164 cells induced some level of antigen-dependent CAR T-cell activation but this was not sufficient to induce cytotoxicity (Fig. 1E). We also asked if T-cell activation markers were induced in the cultures. MUC1 and TnMUC1 CAR T cells had significantly higher expression of T-cell activation markers HLA-DR, CD25, and CD69 2 days after coculture with T47D and OvCAR3 cells (Fig. 1F; Supplementary Fig. S2A and S2B), but there was no significant difference in the expression of T-cell activation markers after coculture with G164 cells. Generally, there was no significant difference in the expression of T-cell exhaustion marker programmed cell death protein 1 (PD1) after coculture with malignant cell lines (Supplementary Fig. S2B). G33, another HGSOC cell line also expressed MUC1 and TnMUC1 (Supplementary Fig. S1D), but was killed only when treated with CAR T cells for a longer period (5 days), suggesting that G33 cells were not as sensitive as OvCAR3 cells to CAR T-cell cytotoxicity (Supplementary Fig. S2C). In summary, in monolayer cultures, we found evidence for a malignant cell–intrinsic resistance to CAR T-cell killing in the G164 cells.

### Impaired death receptor signaling in malignant cells caused resistance to CAR T-cell cytotoxicity

**Figure 2.**
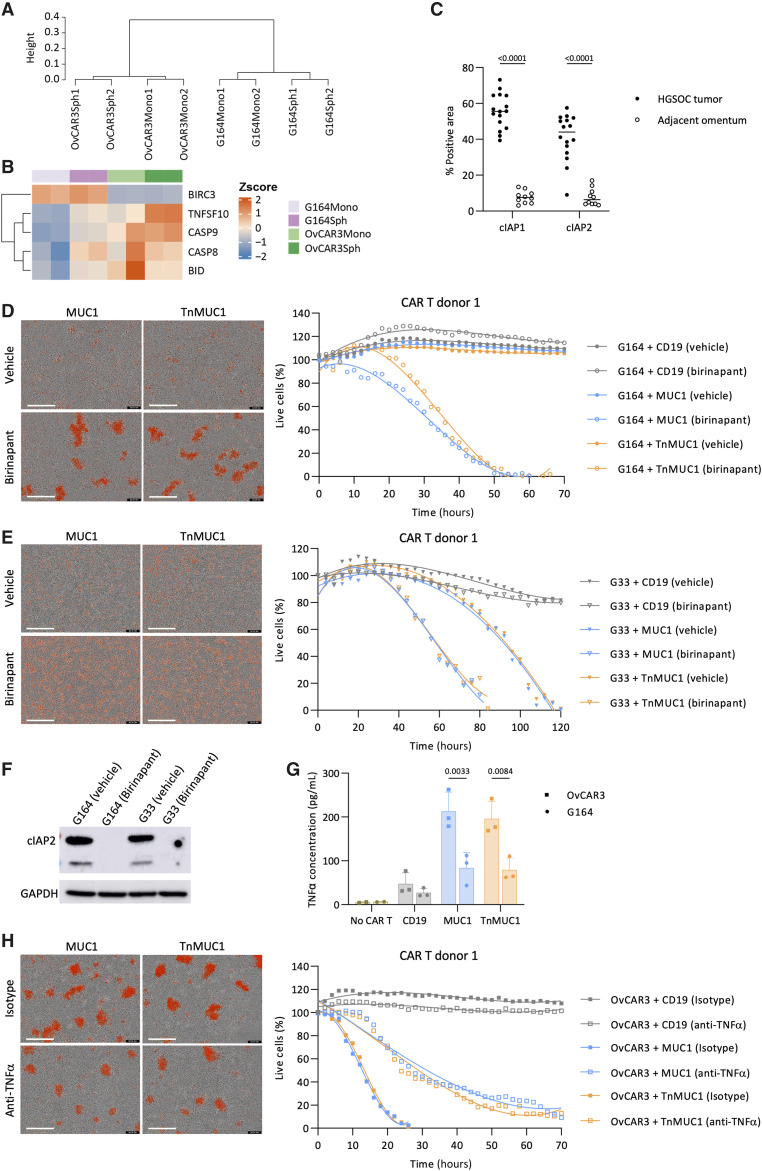
Impaired death receptor signaling in malignant cells caused resistance to CAR T-cell cytotoxicity. **A,** Hierarchical cluster analysis of transcriptomes for OvCAR3 and G164 cells in monolayer and spheroids. **B,** Heatmap illustrating normalized gene expression of differentially expressed genes relating to death receptor signaling in OvCAR3 and G164 cells (adjusted *P* value < 0.05). **C,** cIAP1/2 expression on human HGSOC omental metastasis (*n* = 16) and adjacent omentum (*n* = 10). Statistics performed using two-way ANOVA. **D** and **E,** Representative images (left) and quantification (right) of Incucyte killing assay in which monolayers of G164 (**D**) and G33 (**E**) cells were treated with birinapant and CAR T cells. **F,** Western blot showing the expression of cIAP2 in OvCAR3 and G164 cells treated with birinapant. Representative images of three repeats. **G,** ELISA data showing TNFα concentration after coculturing CAR T cells with monolayers of OvCAR3 and G164 cells for 2 days at 1:5 T:E ratios. Data plotted as mean ± SD for three CAR T-cell donors. Statistics performed using two-way ANOVA. **H,** Representative images (left) and quantification (right) of Incucyte killing assay in which OvCAR3 monolayer was treated with anti-TNFα antibody and CAR T cells. **D–F,** Data shown for one CAR T-cell donor at 1:5 T:E ratio. Images shown are 3 days after treatment. Red, dead cells. Scale bars, 400 μm.

In order to understand the resistance of G164 cells to CAR T cells, we conducted RNA sequencing analysis on G164 and OvCAR3 cells grown in monolayer and as spheroids. Unsupervised clustering showed a clear separation between OvCAR3 and G164 cells (Fig. 2A). Looking at differentially expressed genes related to cell death, we observed distinct differences in the apoptotic gene signatures of OvCAR3 and G164 cells (adjusted *P* value < 0.05, Supplementary Fig. S3A). In particular, the expression of the antiapoptotic molecule *BIRC3* was higher in G164 than in OvCAR3 cells (adjusted *P* value < 0.05, Fig. 2B). Moreover, the expression of proapoptotic death receptor signaling molecules such as *TNFSF10*, *CASP9*, *CASP8*, and *BID* were lower in G164 than OvCAR3 cells (adjusted *P* value < 0.05, Fig. 2B). *BIRC3* encodes for cellular inhibitor of apoptosis protein 2 (cIAP2), which we found to be highly expressed in human HGSOC omental tumors with low levels in adjacent omentum (Fig. 2C).

Recently, it was reported that some patients with leukemia were resistant to CD19 CAR T-cell therapy due to defects in death-receptor signaling (27) and we wondered if this might account for the resistance of G164 cells to the CAR T cells. We therefore treated monolayers of G164 cells with birinapant, a small molecule inhibitor of cIAP1/2. This sensitized G164 cells to CAR T cytotoxicity (Fig. 2D; Supplementary Fig. S3B), which is in accordance with the findings reported in leukemia cells (27). Similarly, G33 cells, which, as we showed above, were not as sensitive as OvCAR3 cells to CAR T-cell cytotoxicity and were killed faster when G33 and CAR T-cell cocultures were treated with birinapant (Fig. 2E; Supplementary Fig. S3C). Treating G164 and G33 cells with birinapant reduced cIAP2 expression (Fig. 2F).

We reasoned that if defects in cell death signaling were contributing to the resistance of G164 cells to CAR T-cell killing, then we should be able to abrogate CAR T-cell cytotoxicity against OvCAR3 cells by inhibiting cell death signaling with agents such as anti- TNF (27). CAR T cells cocultured with OvCAR3 cells produced significantly higher levels of TNFα than CAR T cells co-cultured with G164 cells (Fig. 2G). We cultured OvCAR3 cells with neutralizing antibodies to TNFα and this delayed CAR T-cell cytotoxicity against OvCAR3 cells (Fig. 2H; Supplementary Fig. S3D). Taking these results together, we concluded that malignant cell–intrinsic antigen-independent resistance to CAR T-cell killing might involve impaired death receptor signaling especially involving TNFα pathway. The fact that cIAP1/2 were highly expressed in HGSOC biopsies suggests this might be clinically relevant.

### Primary omental fibroblasts induced CAR T-cell cytotoxicity against G164 cells in suspension spheroids

**Figure 3.**
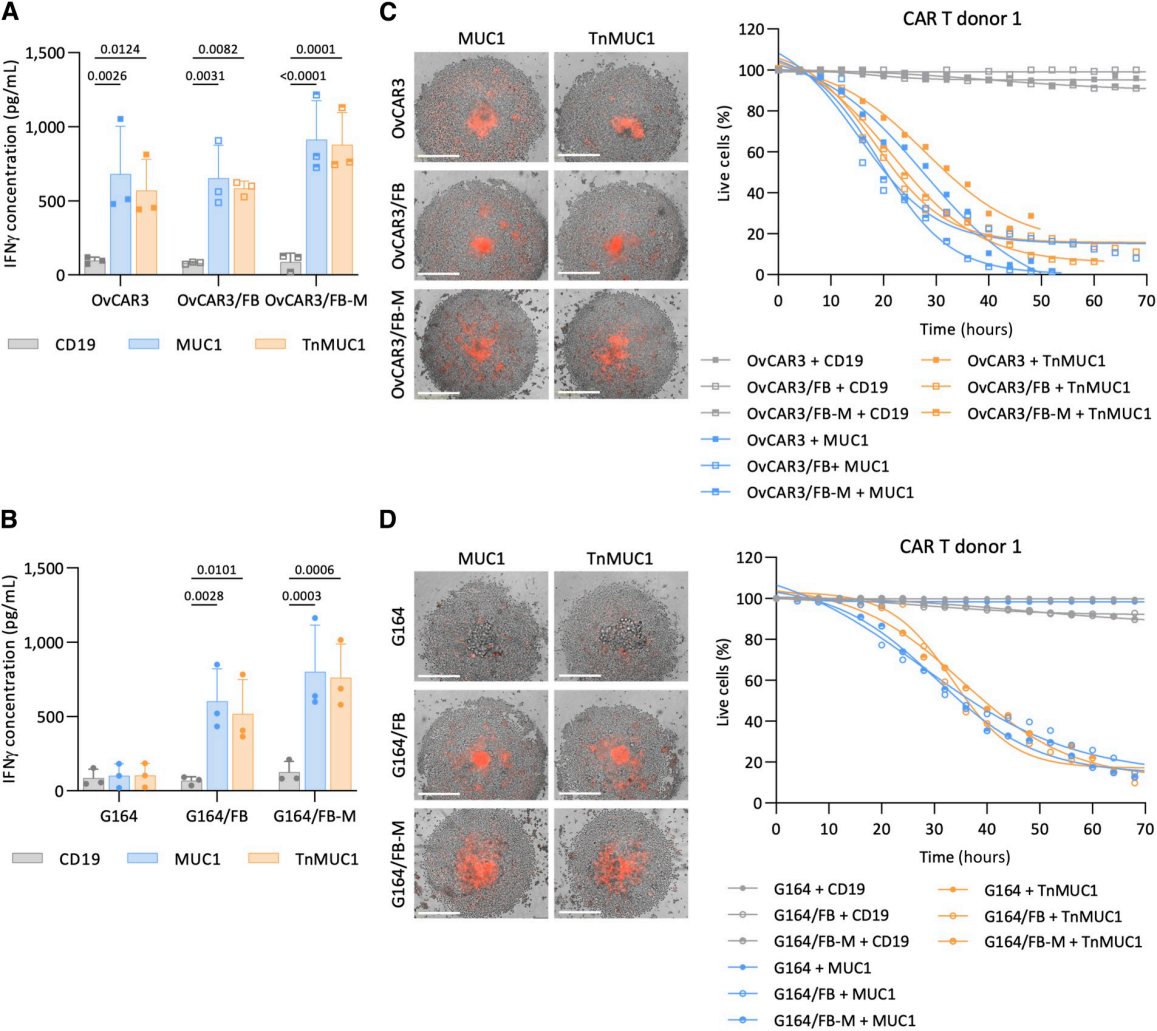
Primary omental fibroblasts induced CAR T-cell cytotoxicity against G164 cells in suspension spheroids. **A** and **B,** ELISA data showing IFNγ concentration after coculturing CAR T cells with OvCAR3 (**A**) and G164 (**B**) spheroids with and without FBs or FB-M for 2 days at 1:5 T:E ratio. Data plotted as mean ± SD for three CAR T-cell donors. Three different fibroblast donors were used for this experiment. Statistics performed using two-way ANOVA. **C** and **D,** Representative images (left) and quantification (right) of Incucyte killing assay in which OvCAR3 (**C**) and G164 (**D**) spheroids with and without FB or FB-M were treated with CAR T cells from one donor at 1:5 T:E ratio. Images shown are 3 days after treatment. Red, dead cells. Scale bars, 400 μm.

As our aim was to study CAR T-cell killing in 3D settings that were more relevant to solid TMEs, we next investigated CAR T-cell activity in spheroid cultures of the two HGSOC cell lines OvCAR3 and G164, this time adding in primary omental FBs isolated from macroscopically normal omentum from patients with gynecological cancer. In a previous publication, we showed that, *in vitro*, these cells express fibroblast activation protein (FAP) and alpha smooth muscle actin (αSMA) and produce ECM proteins such as fibronectin, versican, collagen 1 alpha 1, and collagen 11 alpha 1 (19). MUC1 and TnMUC1 CAR T cells were significantly activated, as measured by IFNγ release, when added to OvCAR3 spheroids with and without FB or FB-conditioned medium (FB-M; Fig. 3A). In line with the findings from monolayer experiments, CAR T cells were not activated when cultured with G164 spheroids (Fig. 3B). However, and unexpectedly, addition of primary omental fibroblasts to G164 spheroids significantly induced IFNγ production by MUC1 and TnMUC1 CAR T cells. This effect could be reproduced by FB-M. We then assessed CAR T-cell cytotoxicity in these culture conditions. CAR T cells from all three donors were cytotoxic against T47D spheroids, but spheroids made from fibroblasts alone were not killed (Supplementary Fig. S4A). OvCAR3 spheroids with and without FB or FB-M were killed by MUC1 and TnMUC1 CAR T cells (Fig. 3C; Supplementary Fig. S4B). Although G164 spheroids were resistant, CAR T cells were cytotoxic against G164 spheroids cultured with FB or FB-M (Fig. 3D; Supplementary Fig. S4C). Replicating the monolayer results described before, treating G164 spheroids with birinapant rendered G164 cells sensitive to MUC1 and TnMUC1 CAR T cells (Supplementary Fig. S4D) and treating OvCAR3 spheroids with anti-TNFα delayed CAR T-cell cytotoxicity (Supplementary Fig. S4E).

### CCL2 produced by fibroblasts activated CCR2/4^+^ CAR T cells to induce antigen-dependent cytotoxicity

**Figure 4.**
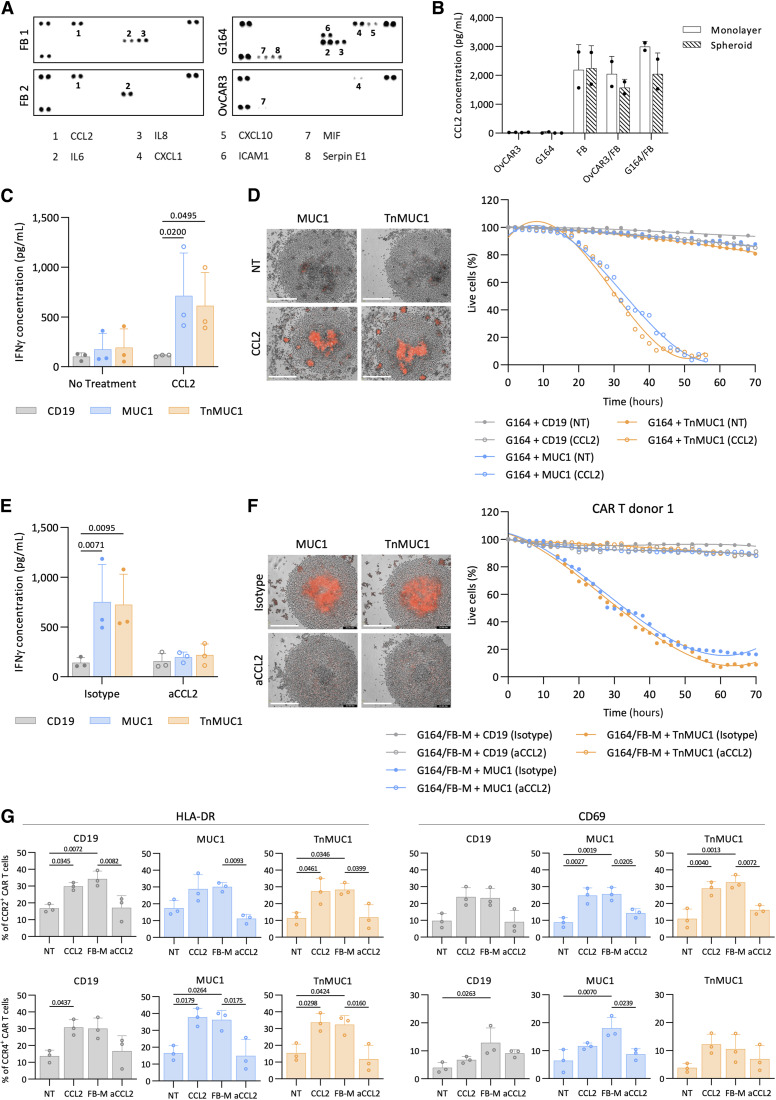
CCL2 produced by fibroblasts activated CCR2/4^+^ CAR T cells to induce antigen-dependent cytotoxicity. **A,** Cytokine profile of FB-, G164-, and OvCAR3-conditioned medium using proteome profiler array. FBs from two donors were used for this experiment. Each cytokine is in duplicate. Dots that are not labeled are reference points. **B,** ELISA showing CCL2 concentrations in monolayers and spheroids of malignant cells with and without FB. Two FB donors were used for this experiment. Data plotted as mean ± SD of two replicates. **C **and** D,** IFNγ concentration measured by ELISA (**C**) and Incucyte CAR T-cell killing assay of G164 spheroids with no treatment (NT) or with recombinant CCL2 (**D**). **E **and** F,** ELISA data showing IFNγ concentration (**E**) and Incucyte killing assay of G164 spheroids cultured in FB-M with anti-CCL2 (aCCL2) antibody (**F**). **G,** Fold change analysis of the expression of T-cell activation markers HLA-DR and CD69 on CCR2^+^ (top) and CCR4^+^ (bottom) CAR T cells after CCL2, FB-M, or FB-M with aCCL2 treatments. **C**, **E**, and **G,** Data plotted as mean ± SD for three CAR T-cell donors. Statistics performed using two-way (**C** and **E**) and one-way ANOVA (**G**). **D** and **F,** Data shown for one CAR T-cell donor. Representative images at day 3 (left) and quantification (right). Red, dead cells. Scale bar, 400 μm.

We then used a proteome profiler cytokine array to identify potential soluble factors produced by the fibroblasts that rendered G164 cells sensitive to CAR T-cell killing (Fig. 4A). Out of the 36 cytokines we tested, only IL6 and C-C motif chemokine ligand 2 (CCL2) were detected in conditioned medium from both primary fibroblast cultures tested. As IL6 was also produced by G164 cells, we hypothesized that CCL2 produced by fibroblasts induced CAR T-cell cytotoxicity against G164 cells. In monolayer and spheroid cultures, malignant cell lines did not produce CCL2 but fibroblasts did (Fig. 4B). Fibroblasts also produced CCL2 in malignant cell/fibroblast cocultures. G164 spheroids significantly activated (Fig. 4C) and induced CAR T-cell cytotoxicity (Fig. 4D; Supplementary Fig. S5A) in the presence of recombinant CCL2. Conversely, addition of an anti-CCL2 antibody abrogated the ability of FB-M to stimulate CAR T-cell activation (Fig. 4E) and cytotoxicity (Fig. 4F; Supplementary Fig. S5B) against G164 spheroids. Furthermore, we knocked down CCL2 in fibroblasts with siRNA (Supplementary Fig. S5C) and cocultured them with G164 spheroids. This delayed CAR T-cell cytotoxicity (Supplementary Fig. S5D), again suggesting CCL2 could influence CAR T-cell cytotoxicity.

CCR2 and CCR4, the receptors for CCL2, were not expressed on the malignant cell lines and fibroblasts (Supplementary Fig. S6A). However, the expression of CCR2 on CAR T cells significantly increased with CCL2 or FB-M treatment (Supplementary Fig. S6B). This was reversed with the addition of anti-CCL2 antibody in FB-M. CAR T cells also had a high CCR4 expression that did not change upon stimulation with recombinant CCL2 or FB-M (Supplementary Fig. S6B). Therefore, we concluded that CCL2 was acting directly on the CAR T cells, and this was supported by increased expression of activation markers HLA-DR and CD69 on CAR T cells treated for 2 days with CCL2 or FB-M (Fig. 4G). This effect was reversed by neutralizing CCL2 in FB-M. There was no significant change in CD25 and PD1 expression on CAR T cells when treated with CCL2 or FB-M (Supplementary Fig. S6C).

Collectively, these data suggested that CCL2 produced by fibroblasts activated CCR2/4^+^ CAR T cells to induce cytotoxic activity. CD19 CAR T cells were also activated by CCL2 and FB-M (Fig. 4G), but these CAR T cells were not cytotoxic against G164 cells (Fig. 4D and F). Therefore, along with the activation of CAR T cells by CCL2, target antigen recognition was also required to induce cytotoxicity against G164 spheroids. Interestingly, monolayers of G164/FB cocultures were not killed by CAR T cells (Supplementary Fig. S6D). This might be because, as shown in Fig. 2B, the expression of proapoptotic molecules was higher in G164 spheroids than in monolayer cultures, thus activation of CAR T cells by CCL2 may have enabled the CAR T cells to overcome the intrinsic G164 resistance in spheroids.

Our results so far have shown that sensitivity of HGSOC malignant cell lines to CAR T cells is governed by both malignant cell–intrinsic and –extrinsic factors; this may be especially relevant to solid tumor microenvironments. In our next experiments, we moved to collagen gels that reproduced some aspects of the ovarian cancer microenvironment (28).

### Dense ECM prevented CAR T-cell migration and cytotoxicity in G164/FB collagen gels

**Figure 5.**
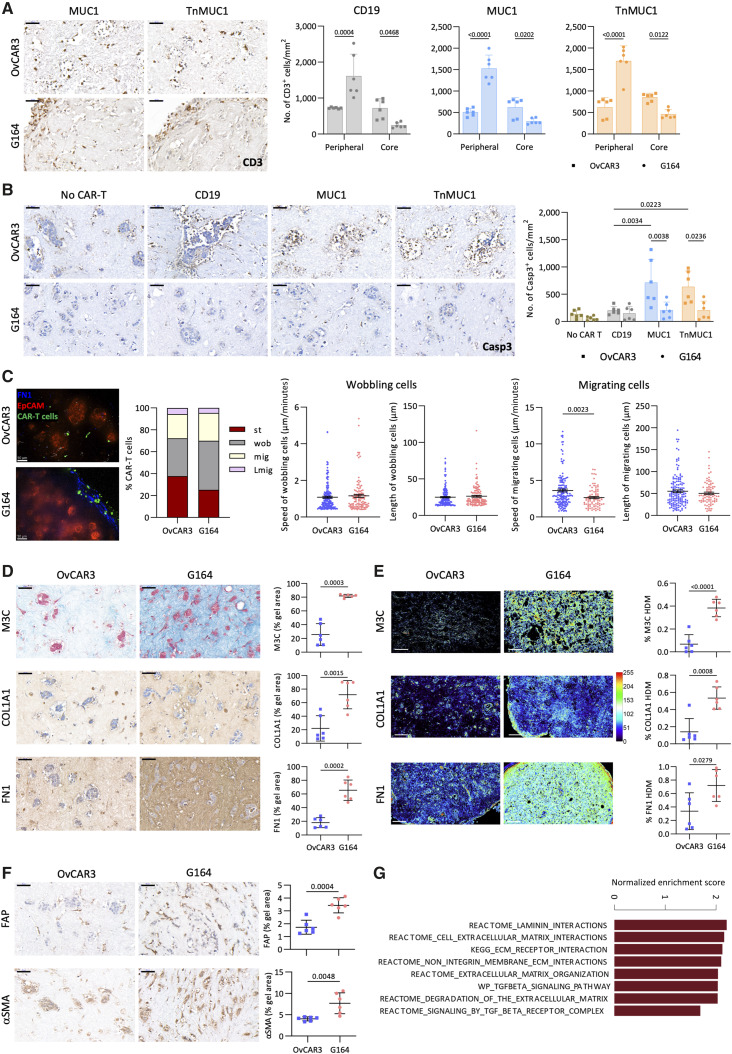
Dense ECM prevented CAR T-cell migration and cytotoxicity in G164/FB collagen gels. **A,** CD3^+^ CAR T cells at the peripheral and core regions of OvCAR3/FB and G164/FB collagen gels. Two different CAR T-cell and FB donors were used in this experiment. **B,** IHC staining and quantification for caspase-3 (Casp3) on OvCAR3/FB and G164/FB collagen gels. **C,** Behavior of CAR T cells in OvCAR3/FB and G164/FB collagen gels. Representative images from three repeats. Blue, fibronectin; green, CAR T cells; red, EpCAM. Scale bar, 50 μm. Proportion of static (st), wobbling (wob), migrating (mig), and long migrating (Lmig) CAR T cells. Speed and length of movement of wobbling (OvCAR3: 172, G164: 112) and migrating (OvCAR3, 145; G164, 83) CAR T cells. **D,** Masson’s trichrome (M3C), COL1A1, and fibronectin (FN1) staining and quantification of OvCAR3/FB and G164/FB collagen gels. **E,** TWOMBLI analysis showing the heatmaps and percentage of HDM for M3C, COL1A1, and FN1 in OvCAR3/FB and G164/FB collagen gels. Scale bar, 100 μm. **F,** Expression of FAP and αSMA on OvCAR3/FB and G164/FB collagen gels. **G,** Barplot illustrating significantly enriched canonical pathways (FDR < 0.05) in G164 vs. OvCAR3 cells in monolayer. **A**, **B**, **D**, and **F,** Data plotted as mean ± SD of three gels per two replicates. Scale bar, 50 μm. Statistics performed using two-way ANOVA (**A** and **B**) and unpaired *t* test (**C–F**).

We cultured the HGSOC cell lines with omental fibroblasts in collagen gels for 14 days and then added CAR T cells for a further 3 days. In G164/FB gels, CD3^+^ CAR T cells were mainly localized at the periphery of the gel (Fig. 5A) and there was no evidence of malignant cell death (Fig. 5B). However, compared to G164/FB gels, OvCAR3/FB gel had significantly higher number of CD3^+^ CAR T cells at the gel core (Fig. 5A) and there was evidence of cell death, as shown by cleaved caspase-3 staining (Fig. 5B). FAP and PAX8 (a transcription factor highly expressed in HGSOC malignant cells; ref. 29) staining showed that only malignant cells, but not fibroblasts, were killed in OvCAR3/FB gels treated with CAR T cells (Supplementary Fig. S7A).

In order to address the dynamic behavior of CAR T cells, we adapted our recently published tissue slicing technique that allows semi-supervised live analysis of immune cell movement (21) to the collagen gels (Supplementary Videos S1 and S2). The proportions of static, wobbling, and migratory CAR T cells were not significantly different in OvCAR3/FB and G164/FB gels (Fig. 5C). Likewise, the speed and length of wobbling CAR T cells were not different. Although the speed of migratory CAR T cells was significantly higher in OvCAR3/FB gels, there was no difference in the length of migration (Fig. 5C). These data suggested that the behavior of CAR T cells did not vary, but as shown above, the localization of CAR T cells varied in OvCAR3/FB and G164/FB gels.

As we had previously shown, fibroblasts secrete ECM in collagen gels when cocultured with malignant cells (19), we wondered whether the resistance of G164 cells in the collagen gels was related to ECM levels. Compared to OvCAR3/FB gels, G164/FB gels had higher levels of ECM content, as measured by collagens, fibronectin, and versican staining (Fig. 5D; Supplementary Fig. S7B). The structure of ECM was then analyzed using TWOMBLI (20), and this showed significantly higher percentage of HDM in G164/FB gels compared to OvCAR3/FB gels (Fig. 5E). Furthermore, the expression of FAP and αSMA was higher in G164/FB gels (Fig. 5F), suggesting that the fibroblasts in these gels had a myofibroblast phenotype, which might have resulted in a denser ECM deposition, thus preventing CAR T-cell migration and cytotoxicity. GSEA performed on differentially expressed genes in monolayers of OvCAR3 and G164 cells revealed that ECM-related and TGFβ-related pathways were significantly enriched in G164 cells (FDR < 0.05, Fig. 5G). In support of this, OvCAR3/FB gels produced significantly lower levels of TGFβ1 compared to G164/FB gels (Supplementary Fig. S7C).

### Inhibition of TGFβ signaling reduced ECM density and stimulated CAR T-cell activity in G164/FB gels

**Figure 6.**
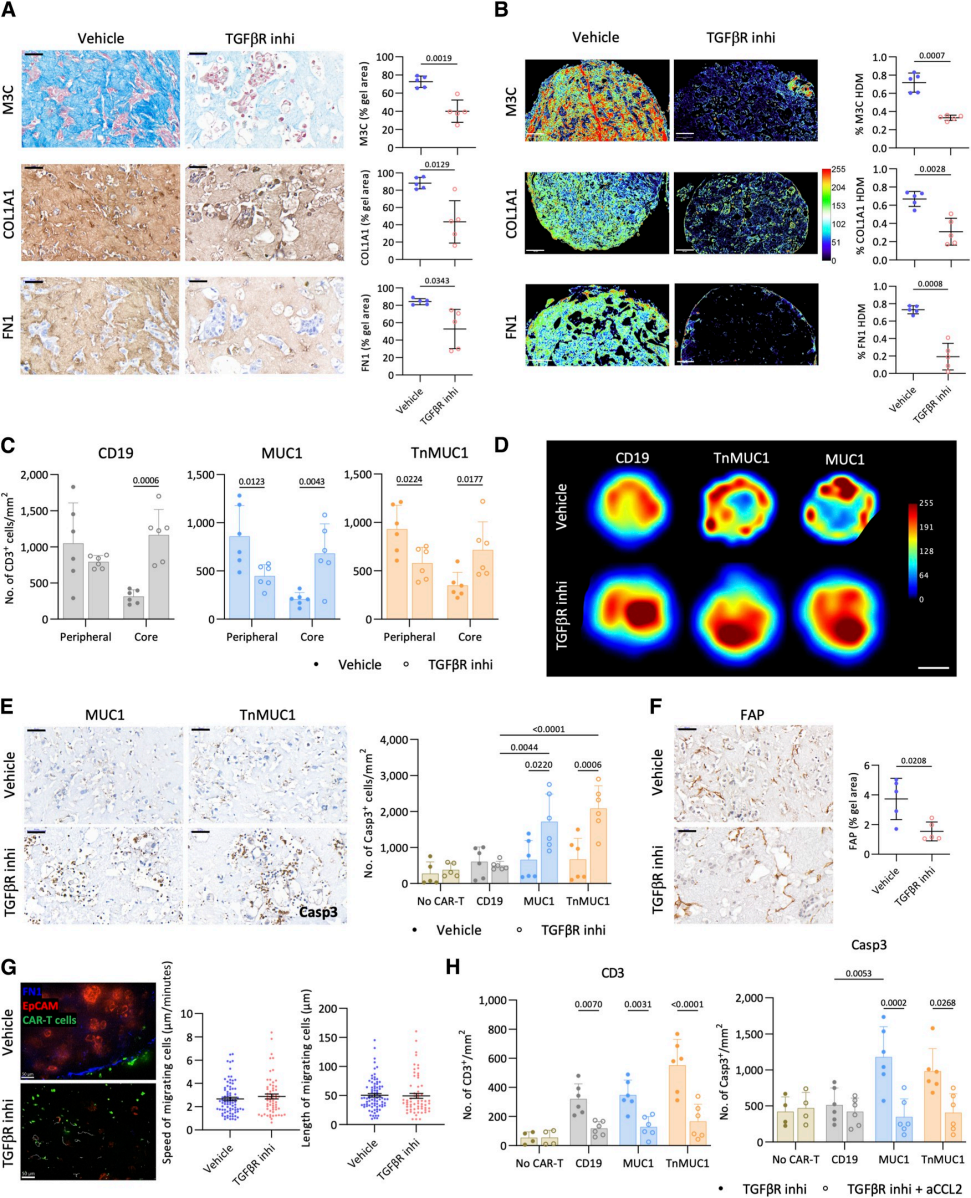
Inhibition of TGFβ signaling reduced ECM density and stimulated CAR T-cell activity in G164/FB gels. **A,** M3C, COL1A1, and FN1 staining and quantification of G164/FB gels treated with TGFβR inhibitor (TGFβR inhi). Scale bar, 50 μm. **B,** TWOMBLI analysis showing the heatmaps and %HDM for M3C, COL1A1, and fibronectin (FN1) in G164/FB gels treated with TGFβR inhibitor. Scale bar, 100 μm. **C,** Number of CD3^+^ CAR T cells at the peripheral and core regions of G164/FB collagen gels treated with TGFβR inhibitor. Two different CAR T-cell and FB donors were used for this experiment. **D,** Heatmaps generated using Definiens Tissue Studio showing the distribution of CAR T cells in G164/FB collagen gels treated with TGFβR inhibitor. Representative images from two/three gels per two replicates. Scale bar, 500 μm. **E,** IHC staining and quantification for caspase-3 (Casp3) on G164/FB gels treated with TGFβR inhibitor. Scale bar, 50 μm. **F,** Expression of FAP on G164/FB gels treated with TGFβR inhibitor. Scale bar, 50 μm. **G,** Behavior of CAR T cells in G164/FB gels treated with TGFβR inhibitor. Representative images from three repeats. Blue, FN1; green, CAR T cells; red, EpCAM. Scale bar, 50 μm. Speed and length of movement of migrating (vehicle, 83; TGFβR inhibitor, 62) CAR T cells. **H,** Number of CD3^+^ (left) and Casp3^+^ (right) on G164/FB gels treated with TGFβR inhibitor and aCCL2. **A–C**, **E**, **F**, and **H,** Data plotted as mean ± SD of two/three gels per two replicates. Statistics performed using unpaired *t* test (**A**, **B**, and **F**) and two-way ANOVA (**C**, **E**, and **H**).

We had previously shown that TGFβ secreted by HGSOC cells acted on fibroblasts to induce ECM deposition in collagen gels (19). Therefore, we reasoned that if ECM levels were important, inhibition of TGFβ signaling may allow CAR T-cell penetration and killing in G164/FB gels. We treated the gels with the TGFβ receptor (TGFβR) inhibitor SB431542 for 14 days and then introduced CAR T cells without TGFβR inhibitor for three further days. Inhibiting TGFβ signaling significantly reduced ECM levels and %HDM in G164/FB gels (Fig. 6A and B; Supplementary Fig. S7D). TGFβR inhibition increased the number of CD3^+^ CAR T cells inside the gels (Fig. 6C). The heatmaps (Fig. 6D) generated using Definiens Tissue Studio showed that without the inhibitor CD3^+^ CAR T cells were mostly distributed at the periphery of the gels, whereas with the TGFβR inhibitor CAR T cells were mainly found at the gel core. This suggested that the inhibition of TGFβ signaling allowed the migration of CAR T cells into G164/FB collagen gels. Moreover, MUC1 and TnMUC1 CAR T cells were cytotoxic in G164/FB gels treated with TGFβR inhibitor (Fig. 6E). FAP staining showed a significant reduction in myofibroblast phenotype in G164/FB gels treated with TGFβR inhibitor (Fig. 6F). Only malignant cells, but not fibroblasts, were killed in G164/FB gels treated with TGFβR inhibitor and CAR T cells (Supplementary Fig. S7E). Collectively these findings suggested that targeting ECM along with CAR T-cell therapy could improve CAR T-cell penetration and activity in solid tumors.

There were no significant differences in the behavior of the CAR T cells in G164/FB gels with and without TGFβR inhibitor (Fig. 6G; Supplementary Fig. S7F; Supplementary Videos S3 and S4), suggesting that reduction in ECM changed the localization of CAR T cells within the gels but not their behavior. We also wanted to see if CCL2 produced by fibroblasts played a role in the collagen gel model. To investigate this, G164/FB gels were treated with TGFβR inhibitor and anti-CCL2 antibody (Fig. 6H). Neutralizing CCL2 significantly reduced the number of CAR T cells in G164/FB gels treated with TGFβR inhibitor, suggesting that CCL2 produced by fibroblasts also acts as a chemoattractant in collagen gels. Moreover, inhibiting CCL2 also abrogated CAR T-cell cytotoxicity in G164/FB gels treated with TGFβR inhibitor (Fig. 6H).

Our results so far led us to conclude that malignant cell–intrinsic factors can impede CAR T-cell killing of some HGSOC cell lines. Fibroblasts can have positive effect on CAR T-cell activity via CCL2 secretion that is counter-balanced by a negative effect by their production of ECM in a TGFβ-dependent manner.

### Vascularized microfluidic chip to investigate CAR T-cell migration and cytotoxicity

**Figure 7.**
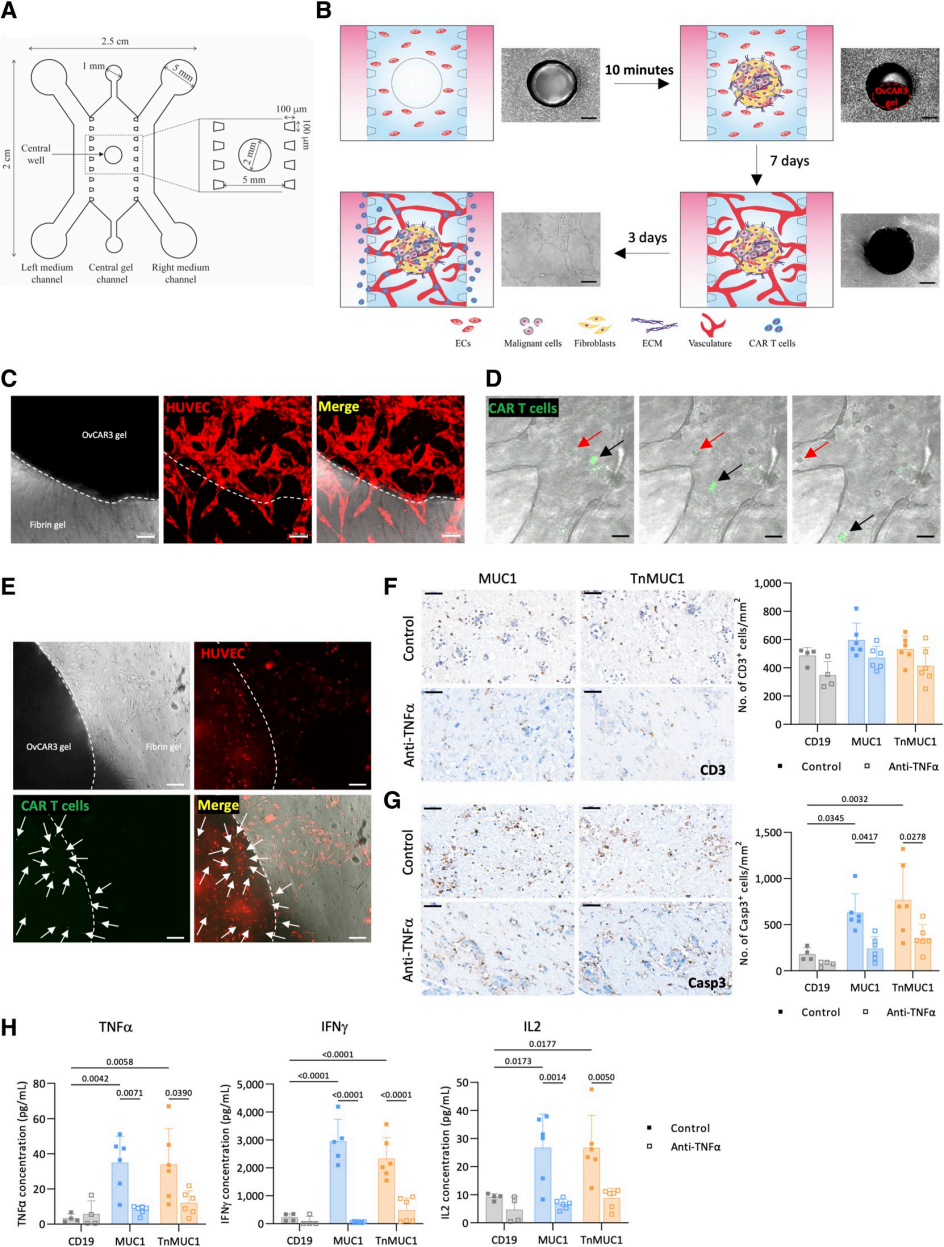
Vascularized microfluidic chip to investigate CAR T-cell migration and cytotoxicity. **A,** Design of the tri-channel microfluidic device with a 2 mm well in the central channel. **B,** Schematic diagram showing the development of ovarian cancer-on-a-chip model. Scale bar, 600 μm. **C,** Immunofluorescence image showing microvasculature in fibrin and OvCAR3 collagen gels (*n* = 3). Red, HUVEC. Scale bar, 100 μm. **D,** Real-time images showing the luminal flow of CAR T cells (arrows) through the vasculature formed within the microfluidic device (*n* = 2). Green, CAR T cells. Scale bar, 20 μm. **E,** Immunofluorescence image showing an ovarian cancer-on-a-chip 3 days after CAR T-cell treatment. CAR T cells (arrows) migrated into the OvCAR3 gel in the middle of the device. Dotted line marks the edge of the central well. Green, CAR T cells; red, HUVEC. Scale bar, 100 μm. **F **and** G,** CD3 (**F**) and caspase‐3 (Casp3; **G**) staining and quantification of OvCAR3 gels isolated from microfluidic device after CAR T-cell and anti-TNFα treatment. **H,** MSD data showing TNFα, IFNγ, and IL2 concentrations on media from microfluidic devices after CAR T-cell and anti-TNFα treatment. **F–H,** Data plotted as mean ± SD of two/three gels per two replicates. Two different CAR T-cell donors were used in this experiment. Scale bar, 50 μm. Statistics performed using two-way ANOVA.

Although we found that CAR T cells could directly penetrate the collagen gels, this did not replicate the systemic delivery of CAR T cells *in vivo*. We therefore developed a vascularized microfluidic chip model to further study CAR T-cell migration and cytotoxicity upon introduction through a microvasculature. The design of the microfluidic chip was adapted from previous publications (30). The device consisted of three parallel channels with a 2 mm diameter well in the central channel (Fig. 7A). We first injected fibrin gel with ECs into the central channel (Fig. 7B). Established collagen gels containing OvCAR3 cells, primary omental fibroblasts, and ECs, cultured for 7 days in a 24-well plate, were then introduced into the well in the central channel. As reported in previous publication (30), in order for the vessels formed within the central channel to penetrate the collagen gel we also added ECs into the collagen gels at 2:2:1 ratio of OvCAR3, FBs, and ECs. As the collagen gel was so dense, it appeared as a dark area in phase contrast images (Fig. 7B). The device was cultured for 7 days, during which, ECs reorganized to form vascular networks in the central channel. CAR T cells were then introduced through the side channels and the device was cultured for additional 3 days (Fig. 7B).

Using a 2 mm biopsy punch we then extracted the collagen gels from the microfluidic devices and subjected them to CD31 staining. This demonstrated the formation of lumenized vasculature within the collagen gels containing ECs (Supplementary Fig. S8A).

OvCAR3 gels isolated from the microfluidic devices expressed Ki67, MUC1, TnMUC1, FAP, and ECM molecules (Supplementary Fig. S8B and S8C). The microvasculature formed within the fibrin gels was able to penetrate the OvCAR3 collagen gel (Fig. 7C; Supplementary Video S5). Furthermore, lumenized microvasculature formed at the interface of the side and the central channels (Supplementary Fig. S8D), allowed the CAR T cells to migrate from the side channel to the vessels formed in the central channel (Supplementary Fig. S8E). A significantly higher number of T cells inside the vasculature suggested that the T cells were transported through the vessels (Supplementary Fig. S8E). The vascular network formed within the microfluidic devices supported the luminal flow of CAR T cells (Fig. 7D; Supplementary Fig. S8E; Supplementary Video S6) and these CAR T cells successfully penetrated the OvCAR3 collagen gel in the center of the device (Fig. 7E).

Once the chip model was established, we asked if the CAR T cells that penetrated the collagen gels were able to kill the OvCAR3 cells. There was no significant difference in the number of CAR T cells that penetrated the collagen gels (Fig. 7F) but there was a significant increase in cell death as measured by caspase-3+ cells in the chips treated with MUC1 or TnMUC1 CAR T cells compared to CD19 CAR T cells (Fig. 7G). We also treated the microfluidic devices with anti-TNFα and CAR T cells to see if death receptor signaling pathways were still important in this more complex model. There was no significant difference in the number of CD3^+^ cells in OvCAR3 gels treated with and without anti-TNFα (Fig. 7F). However, in line with the finding described in avascular models, anti-TNFα significantly reduced CAR T-cell cytotoxicity in OvCAR3 gels (Fig. 7G).

Finally, we measured the levels of proinflammatory cytokines in the effluent from the microfluidic devices using Meso Scale Discovery multiplex kit. The microfluidic cultures produced measurable levels of all cytokines assayed: TNFα, IFNγ, IL1β, IL2, IL4, IL10, and IL12. In cultures treated with anti-TNFα, there was a significant reduction in TNFα, IFNγ, and IL2 concentrations (Fig. 7H), providing further evidence of reduction in CAR T-cell activity by the inhibitor. There were no differences in the levels of IL1β, IL4, IL10, and IL12 (Supplementary Fig. S8F). We concluded that CAR T cells successfully penetrated the collagen gels when delivered through vascularized microfluidic devices and this technology can be used to study factors affecting CAR T-cell activity in solid tumors.

## Discussion

The results of this study suggest that complex 3D human cell models may provide an efficient way of screening multiple cytotoxic human immune cell constructs, allowing us to investigate the interaction between these immune cells and other cells in the TME that could modulate their activity. The models we used are not designed to replace *in vivo* experiments, but our work shows that they are synergistic with mouse models, allowing medium throughput and mechanistic studies to be done with the added advantage of fully human systems. Using 3D *in vitro* human cell models of increasing complexity we have shown that multiple factors could affect CAR T-cell activity within the TME. The further refinement of the 3D models in the microfluidic chip shows the potential for using these human cell models to study factors that influence transport and survival of CAR T cells in the vasculature.

Recent studies have demonstrated the importance of death receptor signaling in malignant cells for T-cell antitumor activity, both in the context of immune checkpoint blockade (ICB) and CAR T cells. Different pieces of evidence support this notion. First, *BIRC2*, encoding for cIAP1, together with cIAP2, encoded by *BIRC3*, are essential checkpoint molecules in preventing TNF receptor 1–mediated cytotoxicity and in *in vitro* and *in vivo* genetic screenings were frequently identified as factors that cause resistance of malignant cells to T-cell–mediated killing (31, 32). Second, nonsynonymous mutations in the TNF signaling pathway in patients with cancer correlated with reduced efficacy of ICB and lower overall survival (31). Third, pharmacologic depletion of cIAP1/2 by birinapant, a bivalent second mitochondria-derived activator of caspases mimetic drug increased the efficiency of ICB in preclinical models of melanoma and glioblastoma (31, 33).

*BIRC2* and other genes required to control death receptor-mediated cytotoxicity were also identified in a genome-wide CRISPR/Cas9 screening whose purpose was to find factors that mediate resistance to CAR T-cell therapy (27, 34). Likewise, Fas-mediated cell death mechanism was critical for the efficacy of CAR T-cell and T-cell bispecific antibody therapies. Diffuse large B‐cell lymphoma patients with high tumoral FAS expression had durable clinical responses and prolonged survival after CD19 CAR T-cell therapy (35). B-cell acute lymphoblastic leukemia (B-ALL) cells from patients who did not respond to CD19 CAR T-cell therapy, even though they exhibited no evidence of antigen loss, had significantly lower death receptor gene signatures than the cells from patients who had complete remission. Furthermore, low death receptor gene signatures correlated with poor overall survival in patients with B-ALL (27). Birinapant was ranked as one of the top three drugs that enhanced CD19 CAR T-cell cytotoxicity against B-ALL cells (34). While cIAP1/2 depletion increased CAR T-cell–mediated malignant cell killing, TNF neutralization impaired CAR T-cell cytotoxicity, suggesting the significance of death receptor–driven apoptotic pathway in CAR T-cell cytotoxicity (27, 34).

We found that the addition of primary human omental fibroblasts to G164 spheroids unexpectedly resulted in CAR T-cell activation. Although most published data suggest an immunosuppressive role of fibroblasts in the TME, there are few studies that described the immunostimulatory effects. IL6 produced by cancer-associated fibroblasts (CAF) augmented the production of proinflammatory cytokines such as IFNγ and IL17A by tumor-infiltrating T cells (36). Likewise, cocultures of murine CAFs and dendritic cells activated T cells to produce IFNα and IFNγ and induce cytotoxicity against malignant cells *in vitro* and *in vivo* (37). In non–small cell lung cancer the presence of CAFs that express meflin correlated with retained vascularization and CD4^+^ T-cell infiltration (38). Meflin+ CAFs improved ICB responses in murine xenograft models (38). Furthermore, 40% of patients with non–small cell lung cancer with high meflin+ CAFs responded to ICB with significant improvement in overall and progression-free survival (38). The depletion of αSMA+ myofibroblasts in PDAC mouse models promoted an immunosuppressive TME and aggressive cancer phenotype with poor animal survival (39).

Our experiments revealed that CCL2 produced by fibroblasts stimulated CAR T-cell activation and cytotoxicity against G164 spheroids. CCL2 is a potent chemoattractant for CCR2/4^+^ immune cells (40). Introducing CCR2 expression on CAR T cells enhanced trafficking, infiltration, and homing of CAR T cells at the tumor sites (41, 42). Independent to the chemotactic function of CCL2, our data suggest that CCL2 activated CCR2/4^+^ CAR T cells, thus promoting cytotoxicity against malignant cells. This mechanism is not widely reported in the literature. However, there are evidence that CCL2 significantly enhanced the production of proinflammatory cytokines IFNγ and IL17A, upregulated the expression of cytotoxic degranulation marker CD107A and promoted cytotoxicity of CAR T cells (41, 42).

Most studies have investigated the effect of CAR T cells in 2D cultures and murine models of solid tumors. To date, there are only few studies that reported CAR T-cell activity in 3D models ranging from spheroids to microfluidic devices (43–48). These studies have shown CAR T-cell migration, infiltration, cytotoxicity, and production of proinflammatory cytokines were limited in 3D models as compared to 2D cultures (45–47). However, majority of the 3D models used to study CAR T cells only contain malignant cells and are criticized for lacking CAFs or other stromal cells. Our findings demonstrated that multiple interactions between malignant cells, CAFs and CAR T cells affect the efficacy of CAR T-cell therapy in solid tumors. In our 3D model, we have previously published that TGFβ secreted by HGSOC cell lines stimulated fibroblasts to produce ECM (19). This dense ECM prevented CAR T-cell infiltration into collagen gels. High collagen density significantly reduced T-cell proliferation, increased the ratio of CD4 to CD8 T cells, downregulated markers of cytotoxic activity, and upregulated markers of regulatory activity (49). Furthermore, T-cell migration was limited in ECM-dense areas of human lung tumors (50). This highlights the need for 3D multicellular models that recapitulate vital features of the TME, such as ECM, to study CAR T-cell activity *in vitro*.

Three-dimensional *in vitro* human cell models can be manipulated by adding and removing cellular or noncellular components of the TME to investigate specific cell–cell and cell–matrix interactions that could influence therapy efficacy. Using complex microfluidic models, it is possible to replicate tissue interfaces and physiologically relevant mechanical conditions. In this study, we utilized the power of 3D *in vitro* models to investigate factors affecting CAR T activity in solid tumors, thus demonstrating that multicellular 3D models may accelerate the development of immune therapy in solid tumors. Having established that four cell types can be cultured together and shown that they can be used to identify factors that impact on CAR T-cell activity, the next steps will be to increase complexity of the models to study the influence of other cell types. For instance, although our data suggest that CCL2 could activate CAR T cells, CCL2 is also important for regulating other cells such as macrophages, which can exhibit immunosuppressive effects. Therefore, 3D models comprising macrophages could help elucidate the effect of this cell type on CAR T-cell function.

## Supplementary Material

Supplementary Video 1 Behavior of CAR-T cells in OvCAR3/FB gels

Supplementary Video 2 Behavior of CAR-T cells in G164/FB gels

Supplementary Video 3 Behavior of CAR-T cells in G164/FB gels without TGFβ-R inhi

Supplementary Video 4 Behavior of CAR-T cells in G164/FB gels with TGFβ-R inhi

Supplementary Video 5 Microvasculature formed within fibrin gel penetrated the OvCAR3 collagen gel

Supplementary Video 6 Luminal flow of CAR-T cells through vessels formed in the microfluidic device

Supplementary Figure 1 OvCAR3 cells were sensitive but G164 cells were resistant to CAR-T cell cytotoxicity in monolayer cultures.

Supplementary Figure 2 OvCAR3 cells were sensitive but G164 cells were resistant to CAR-T cell cytotoxicity in monolayer cultures.

Supplementary Figure 3 Impaired death receptor signaling in malignant cells caused resistance to CAR-T cell cytotoxicity.

Supplementary Figure 4 Primary omental fibroblasts induced CAR-T cell cytotoxicity against G164 cells in suspension spheroids.

Supplementary Figure 5 CCL2 produced by fibroblasts activated CCR2/4+ CAR-T cells to induce antigen-dependent cytotoxicity.

Supplementary Figure 6 CCL2 produced by fibroblasts activated CCR2/4+ CAR-T cells to induce antigen-dependent cytotoxicity.

Supplementary Figure 7 Malignant cells and fibroblasts co-culture collagen gels treated with CAR-T cells.

Supplementary Figure 8 Vascularized microfluidic chip to investigate CAR-T cell migration and cytotoxicity.

Supplementary Table 1 Fluorophore-conjugated antibodies used for flow cytometry.

Supplementary Table 2 Primary antibodies used for immunohistochemistry.

Supplementary Videos legend Legend for supplementary videos 1-6
