# Supplementary material for: Human 3D Ovarian Cancer Models Reveal Malignant Cell–Intrinsic and –Extrinsic Factors That Influence CAR T-cell Activity
Source: Cancer Res. 2024 May 31;84(15):2432–49. doi: 10.1158/0008-5472.CAN-23-3007 (PMC11292204; doi:10.1158/0008-5472.CAN-23-3007)
Supplement: Supplementary Figure 1 — OvCAR3 cells were sensitive but G164 cells were resistant to CAR-T cell cytotoxicity in monolayer cultures. [file can-23-3007_supplementary_figure_1_suppsf1.pdf]

Supplementary Figure 1

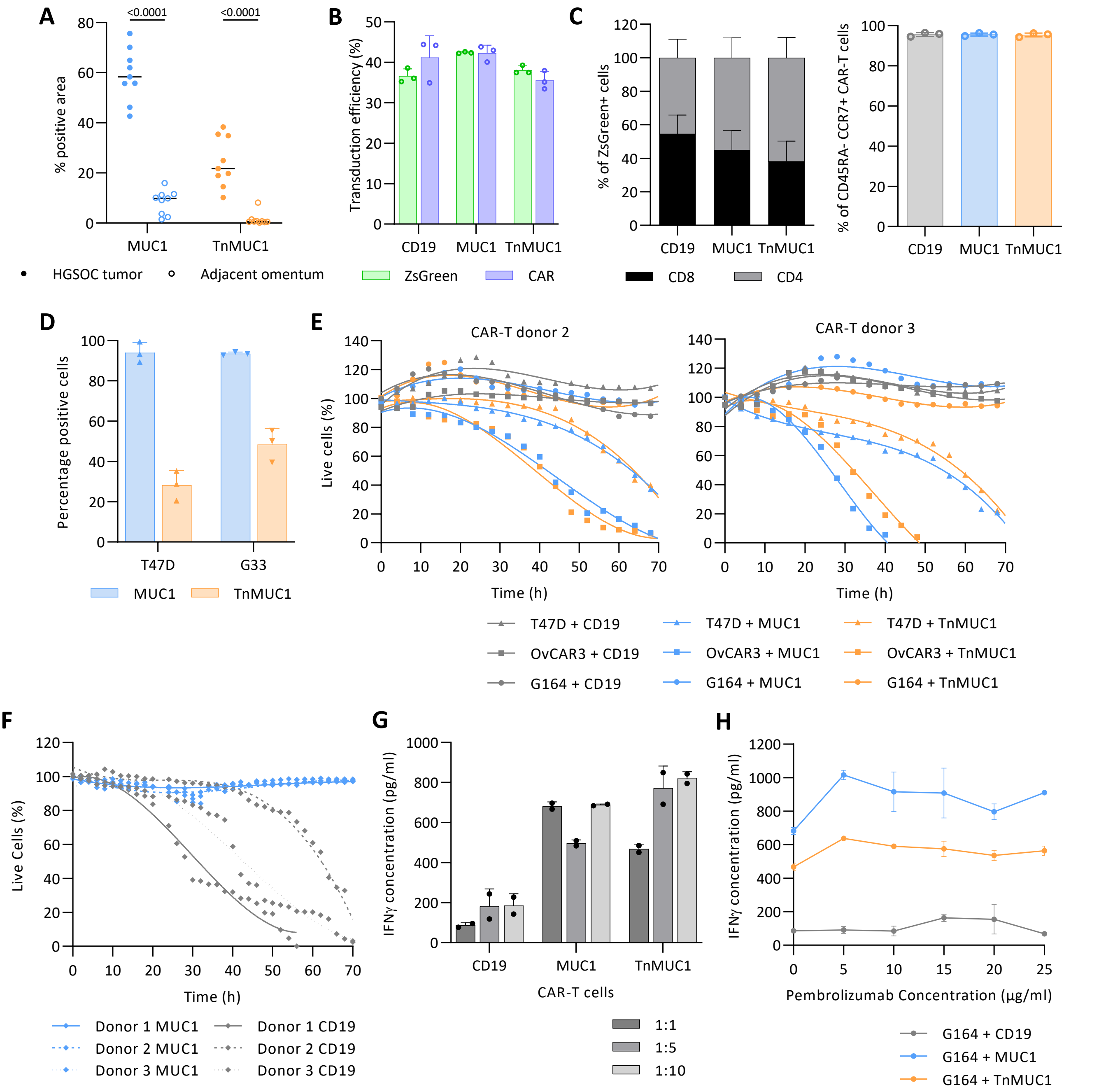

Supplementary Figure 1: OvCAR3 cells were sensitive but G164 cells were resistant to CAR-T cell cytotoxicity in monolayer cultures. (A) MUC1 and TnMUC1 expression on human HGSOC omental metastasis (n=9) and adjacent omentum (n=9). Statistics performed using 2-way ANOVA. **(B)** Transduction efficiency of CAR-T cells assessed by FC. Data plotted as mean ± SD for three CAR-T cell donors. **(C)** FC analysis of CD8+ and CD4+ CAR-T cells (left panel) and central memory phenotype of CAR-T cells (right panel). Data plotted as mean ± SD for three CAR-T cell donors. **(D)** FC analysis of the expression of MUC1 and TnMUC1 on monolayers of T47D and G33 cells. Data plotted as mean ± SD of three replicates. **(E)** Incucyte killing assay in which monolayers of malignant cell lines were treated with CAR-T cells from two different donors at 1:5 T:E ratio. **(F)** Incucyte killing assay of NALM6 cells treated with CD19 CAR-T cells from three donors at 1:5 T:E ratio. **(G & H)** IFNγ concentration measured by MSD after co-culturing CAR-T cells with G164 cells for two days **(G)** at decreasing T:E ratios and **(H)** varying concentrations of pembrolizumab. Data plotted as mean ± SD of two technical replicates using a single CAR-T cell donor.
