# Supplementary material for: Human 3D Ovarian Cancer Models Reveal Malignant Cell–Intrinsic and –Extrinsic Factors That Influence CAR T-cell Activity
Source: Cancer Res. 2024 May 31;84(15):2432–49. doi: 10.1158/0008-5472.CAN-23-3007 (PMC11292204; doi:10.1158/0008-5472.CAN-23-3007)
Supplement: Supplementary Figure 2 — OvCAR3 cells were sensitive but G164 cells were resistant to CAR-T cell cytotoxicity in monolayer cultures. [file can-23-3007_supplementary_figure_2_suppsf2.pdf]

Supplementary Figure 2

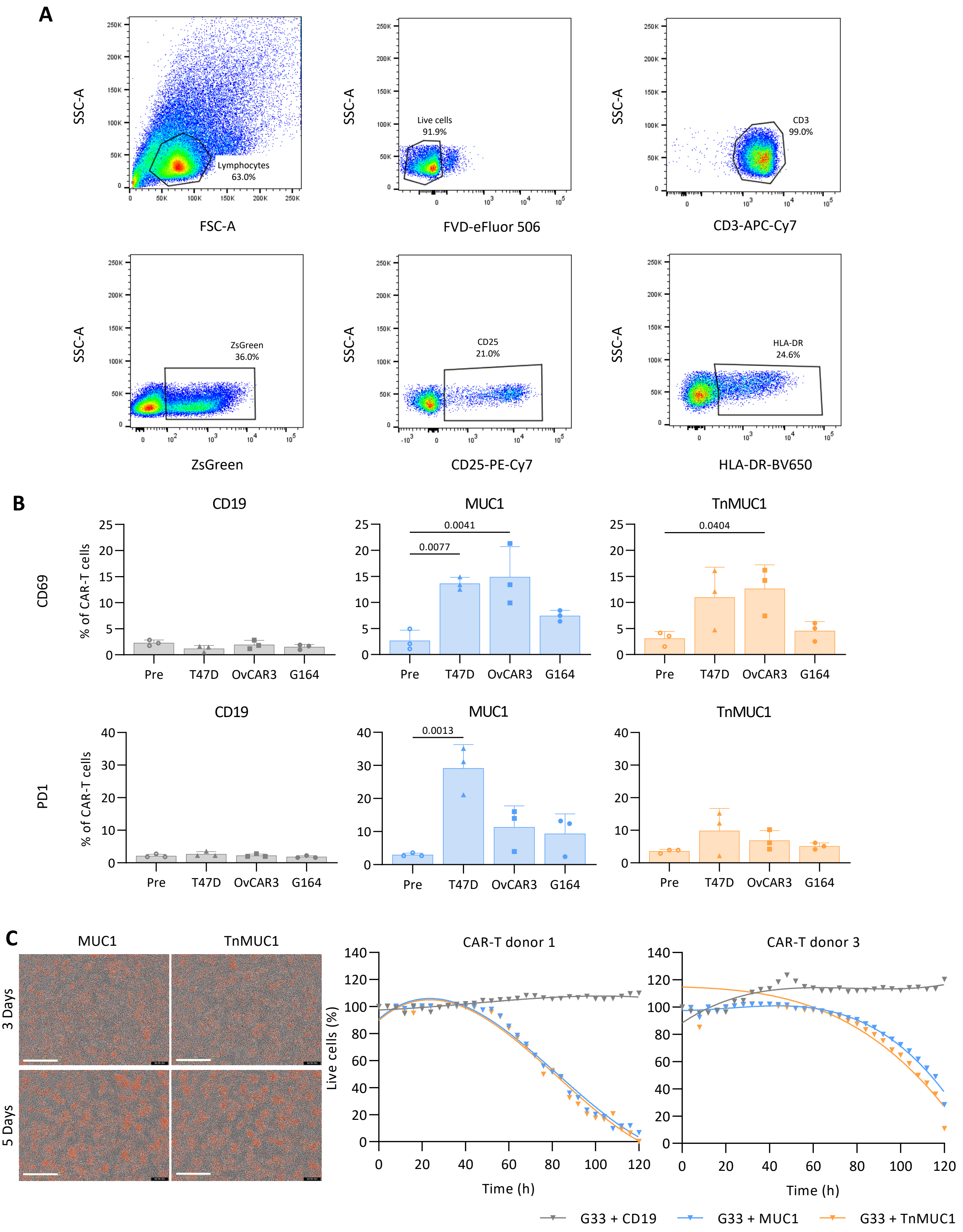

Supplementary Figure 2: OvCAR3 cells were sensitive but G164 cells were resistant to CAR-T cell cytotoxicity in monolayer cultures. (A) FC gating strategy for CAR-T cells after co-culture with target cell lines. Dot plots displays representative example of samples sorted for single cells (SSC-A vs FSC-A), live cells, CD3+, ZsGreen+ CAR-T cells that express relevant markers. **(B)** FC analysis of the expression of CD69 and PD1 on CAR-T cells pre and two days post co-culture with malignant cell lines. Data plotted as mean ± SD for three CAR-T cell donors. Statistics performed using 1-way ANOVA. **(C)** Representative images (left panel) and quantification (right panel) of Incucyte killing assay in which monolayer of G33 cells was treated with CAR-T cells from two donors. Images shown are three and five days after treatment. Scale bars: 400 µm.
