# Supplementary material for: Human 3D Ovarian Cancer Models Reveal Malignant Cell–Intrinsic and –Extrinsic Factors That Influence CAR T-cell Activity
Source: Cancer Res. 2024 May 31;84(15):2432–49. doi: 10.1158/0008-5472.CAN-23-3007 (PMC11292204; doi:10.1158/0008-5472.CAN-23-3007)
Supplement: Supplementary Figure 3 — Impaired death receptor signaling in malignant cells caused resistance to CAR-T cell cytotoxicity. [file can-23-3007_supplementary_figure_3_suppsf3.pdf]

Supplementary Figure 3

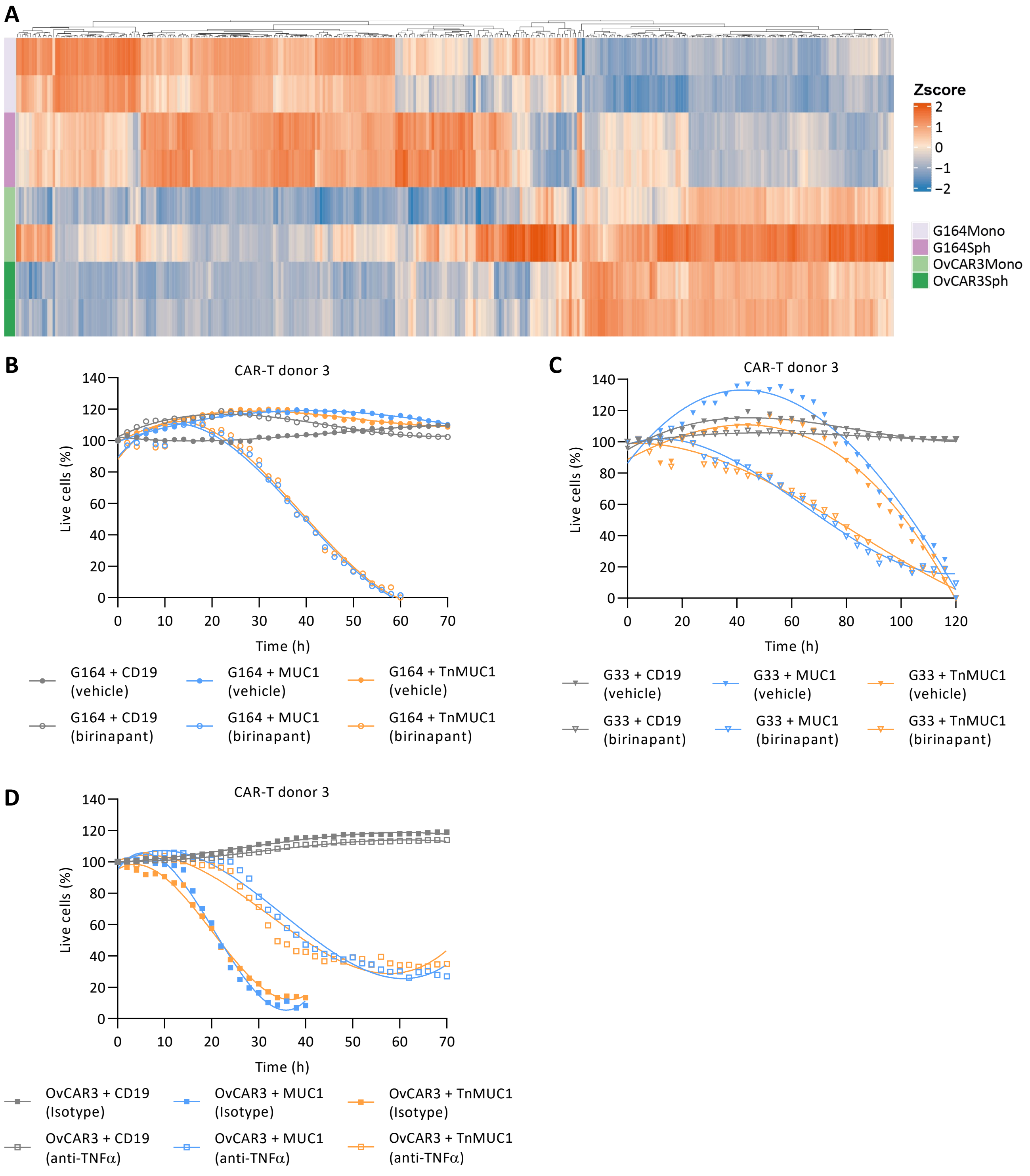

Supplementary Figure 3: Impaired death receptor signaling in malignant cells caused resistance to CAR-T cell cytotoxicity. (A) Heatmap of normalized gene expression for all the differentially expressed apoptosis-related genes in monolayer and spheroids of OvCAR3 and G164 cells (adjusted p-value < 0.05). **(B & C)** Incucyte killing assay in which monolayers of **(B)** G164 and **(C)** G33 cells were treated with birinapant and CAR-T cells from another donor at 1:5 T:E ratio. **(D)** Incucyte killing assay in which OvCAR3 monolayer was treated with anti-TNFα antibody and CAR-T cells from another donor at 1:5 T:E ratio.
