# Supplementary material for: Human 3D Ovarian Cancer Models Reveal Malignant Cell–Intrinsic and –Extrinsic Factors That Influence CAR T-cell Activity
Source: Cancer Res. 2024 May 31;84(15):2432–49. doi: 10.1158/0008-5472.CAN-23-3007 (PMC11292204; doi:10.1158/0008-5472.CAN-23-3007)
Supplement: Supplementary Figure 4 — Primary omental fibroblasts induced CAR-T cell cytotoxicity against G164 cells in suspension spheroids. [file can-23-3007_supplementary_figure_4_suppsf4.pdf]

Supplementary Figure 4

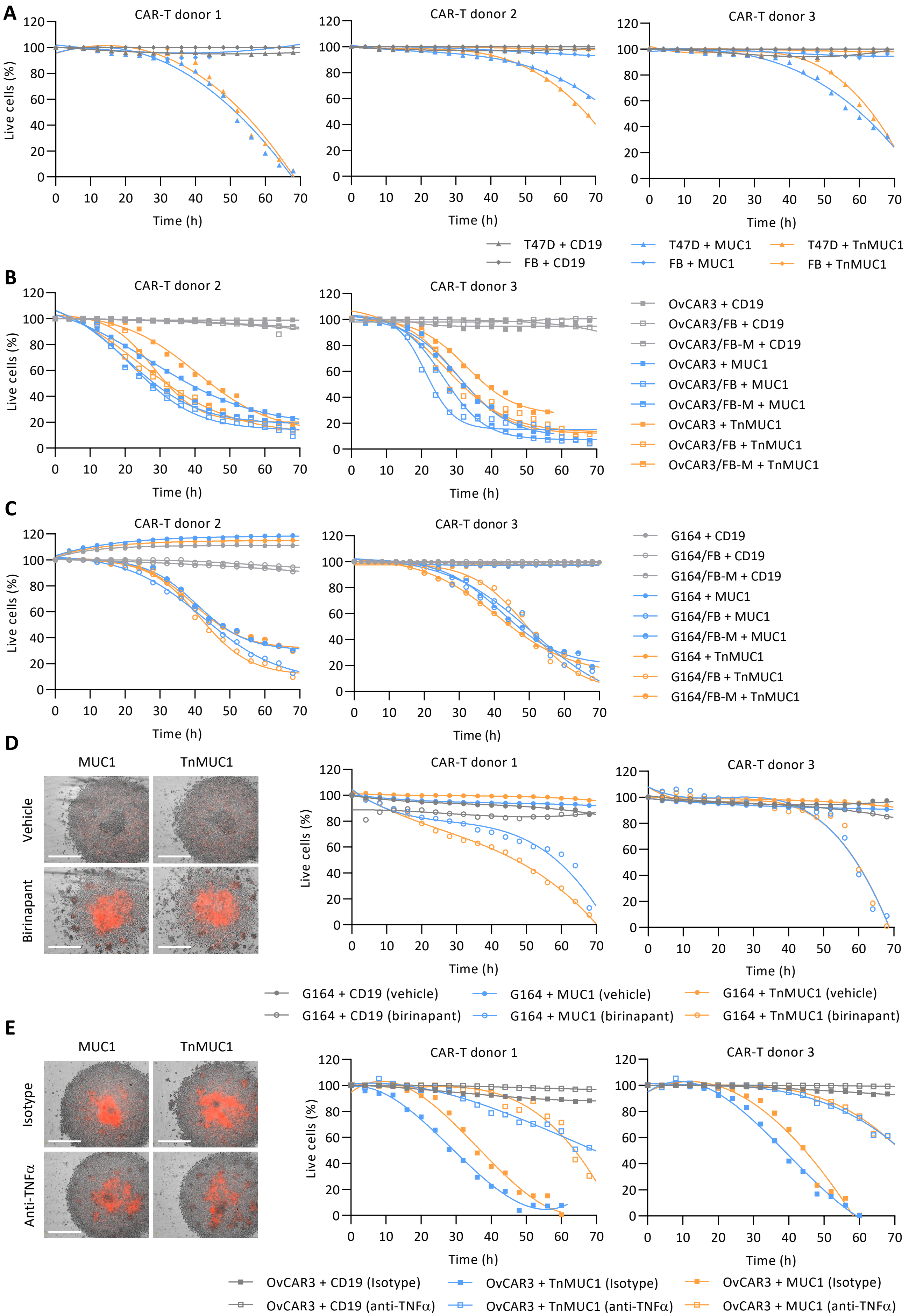

Supplementary Figure 4: Primary omental fibroblasts induced CAR-T cell cytotoxicity against G164 cells in suspension spheroids. **(A)** Incucyte killing assay for T47D and primary omental fibroblasts (FB) spheroids treated with CAR-T cells from three different donors at 1:5 T:E ratio. **(B & C)** Incucyte killing assay in which **(B)** OvCAR3 and **(C)** G164 spheroids with and without FB or fibroblast-conditioned media (FB-M) were treated with CAR-T cells from two different donors at 1:5 T:E ratio. Different fibroblast donors were using for each experiment. **(D & E)** Representative images (left panel) and quantification (right panel) of Incucyte killing assay for **(D)** G164 spheroids treated with birinapant and **(E)** OvCAR3 spheroids treated with anti-TNFα and CAR-T cells from two different donors at 1:5 T:E ratio. Images shown are three days after treatment. Red = dead cells. Scale bars: 400 µm.
