# Supplementary material for: Human 3D Ovarian Cancer Models Reveal Malignant Cell–Intrinsic and –Extrinsic Factors That Influence CAR T-cell Activity
Source: Cancer Res. 2024 May 31;84(15):2432–49. doi: 10.1158/0008-5472.CAN-23-3007 (PMC11292204; doi:10.1158/0008-5472.CAN-23-3007)
Supplement: Supplementary Figure 5 — CCL2 produced by fibroblasts activated CCR2/4+ CAR-T cells to induce antigen-dependent cytotoxicity. [file can-23-3007_supplementary_figure_5_suppsf5.pdf]

Supplementary Figure 5

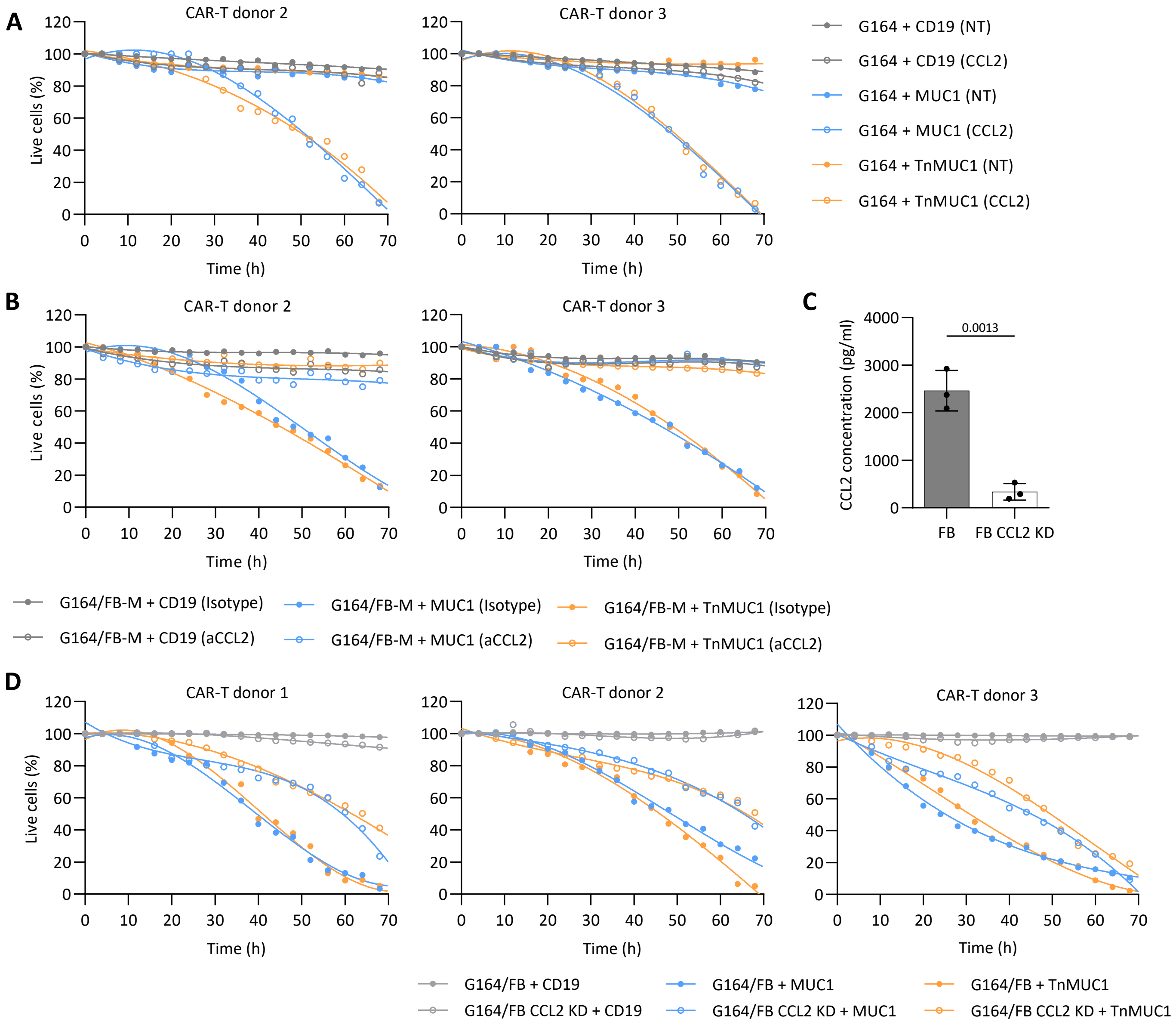

Supplementary Figure 5: CCL2 produced by fibroblasts activated CCR2/4+ CAR-T cells to induce antigen-dependent cytotoxicity. (A & B) Incucyte killing assay in which G164 spheroids cultured with **(A)** recombinant CCL2 and **(B)** anti-CCL2 antibody (aCCL2) in FB-M were treated with CAR-T cells from two different donors at 1:5 T:E ratio. **(C)** ELISA showing CCL2 concentration in CCL2 knockdown (KD) FB two days after siRNA transfection. Data plotted as mean ± SD for three FB donors. Statistics performed using unpaired t test. **(D)** Quantification of Incucyte killing assay in which G164 spheroids co-cultured with CCL2 KD FB were treated with CAR-T cells from three donor at 1:5 T:E ratio.
