# Supplementary material for: Human 3D Ovarian Cancer Models Reveal Malignant Cell–Intrinsic and –Extrinsic Factors That Influence CAR T-cell Activity
Source: Cancer Res. 2024 May 31;84(15):2432–49. doi: 10.1158/0008-5472.CAN-23-3007 (PMC11292204; doi:10.1158/0008-5472.CAN-23-3007)
Supplement: Supplementary Figure 6 — CCL2 produced by fibroblasts activated CCR2/4+ CAR-T cells to induce antigen-dependent cytotoxicity. [file can-23-3007_supplementary_figure_6_suppsf6.pdf]

Supplementary Figure 6

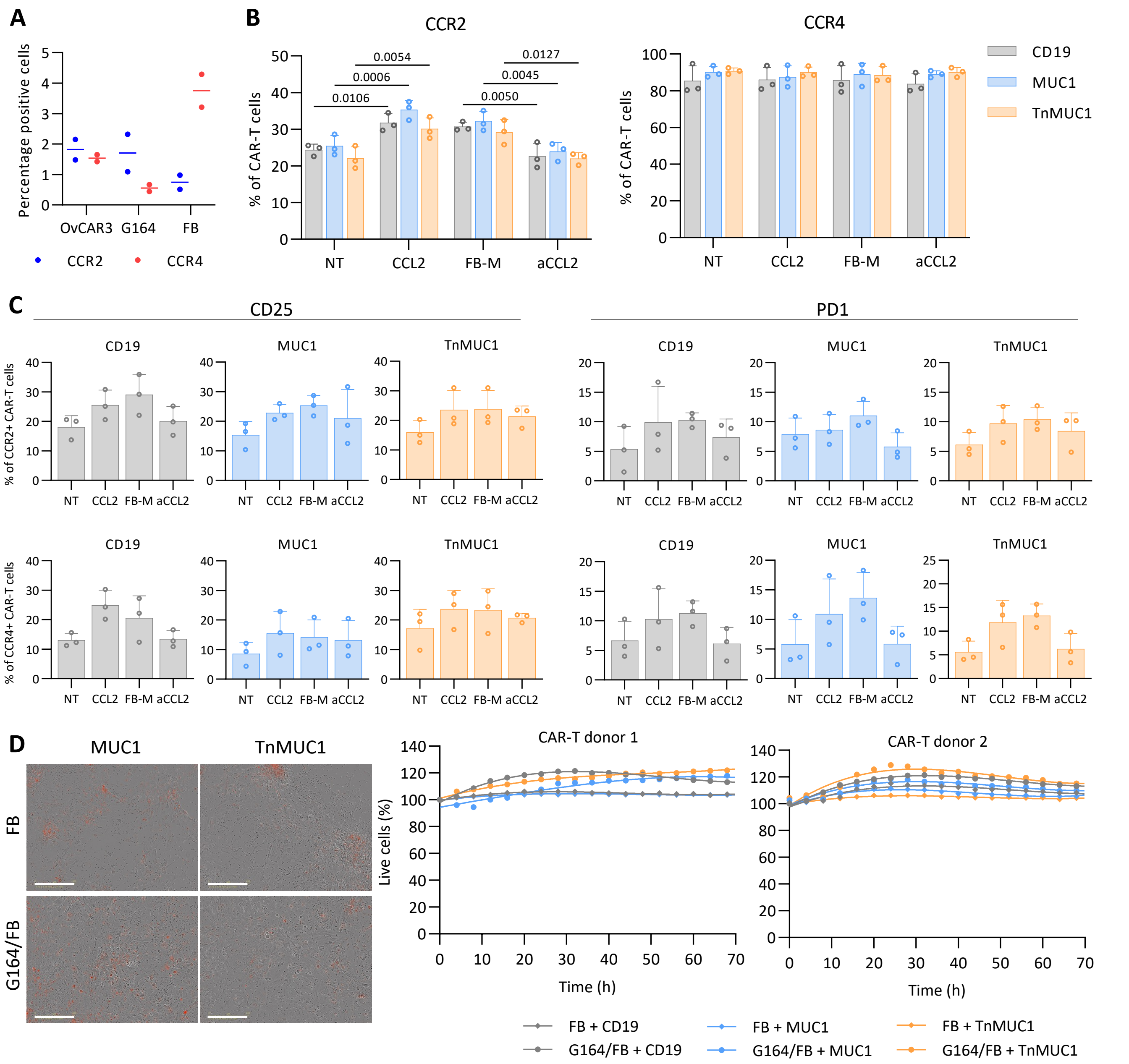

Supplementary Figure 6: CCL2 produced by fibroblasts activated CCR2/4+ CAR-T cells to induce antigen-dependent cytotoxicity. (A) CCR2/4 expression on malignant cells and two different fibroblast donors. **(B)** Expression of CCR2 (left panel) and CCR4 (right panel) on CAR-T cells after CCL2, FB-M or FB-M with aCCL2 treatments. Statistics performed using 2-way ANOVA. **(C)** Expression of T cell activation marker CD25 and exhaustion marker PD1 on CCR2+ (top panel) and CCR4+ (bottom panel) CAR-T cells after CCL2, FB-M or FB-M with aCCL2 treatments. **(B & C)** Data plotted as mean ± SD for three CAR-T cell donors. **(D)** Representative images (left panel) and quantification (right panel) of Incucyte killing assay in which monolayer of G164/FB co-cultures were treated with CAR-T cells from two donors at 1:5 T:E. Images shown are three days after treatment. Red = dead cells. Scale bars: 400 µm.
