# Supplementary material for: Human 3D Ovarian Cancer Models Reveal Malignant Cell–Intrinsic and –Extrinsic Factors That Influence CAR T-cell Activity
Source: Cancer Res. 2024 May 31;84(15):2432–49. doi: 10.1158/0008-5472.CAN-23-3007 (PMC11292204; doi:10.1158/0008-5472.CAN-23-3007)
Supplement: Supplementary Figure 7 — Malignant cells and fibroblasts co-culture collagen gels treated with CAR-T cells. [file can-23-3007_supplementary_figure_7_suppsf7.pdf]

Supplementary Figure 7

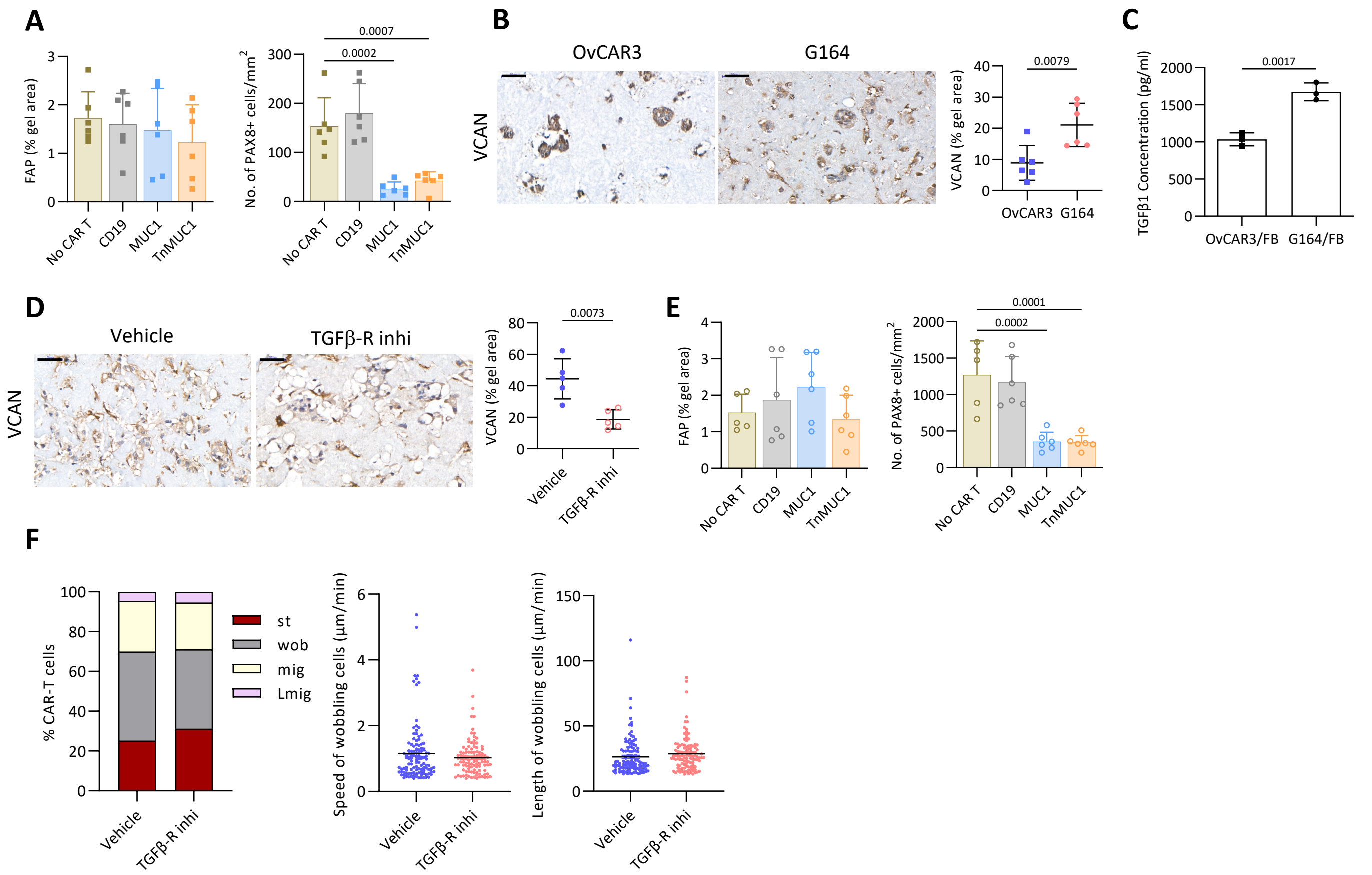

Supplementary Figure 7: Malignant cells and fibroblasts co-culture collagen gels treated with CAR-T cells. (A) Quantification of FAP (left panel) and PAX8 (right panel) on OvCAR3/FB gels treated with CAR-T cells. **(B)** Versican (VCAN) staining and quantification on OvCAR3/FB and G164/FB collagen gels. Scale bar: 50 µm. **(C)** TGFβ1 concentration in OvCAR3/FB and G164/FB collagen gels. Data plotted as mean ± SD for three replicates. Three different FB donors were used for in this experiment. **(D)** VCAN staining on G164/FB gels treated with TGFβ receptor inhibitor (TGFβ-R inhi). Scale bar: 50 µm. **(E)** IHC quantification of FAP (left panel) and PAX8 (right panel) on G164/FB gels treated with TGFβ-R inhi for fourteen days and then treated with CAR-T cells for three further days without TGFβ-R inhi. **(A, B, D, E)** Data plotted as mean ± SD for two/three gels per two replicates. **(F)** Proportion of static (st), wobbling (wob), migrating (mig) and long migrating (Lmig) CAR-T cells in G164/FB gels treated with TGFβ-R inhi. Speed and length of movement of wobbling (vehicle: 112, TGFβ-R inhi: 108) CAR-T cells. Statistics performed using **(A & E)** 2-way ANOVA and **(B-D)** unpaired t test.
