# Supplementary material for: Human 3D Ovarian Cancer Models Reveal Malignant Cell–Intrinsic and –Extrinsic Factors That Influence CAR T-cell Activity
Source: Cancer Res. 2024 May 31;84(15):2432–49. doi: 10.1158/0008-5472.CAN-23-3007 (PMC11292204; doi:10.1158/0008-5472.CAN-23-3007)
Supplement: Supplementary Figure 8 — Vascularized microfluidic chip to investigate CAR-T cell migration and cytotoxicity. [file can-23-3007_supplementary_figure_8_suppsf8.pdf]

Supplementary Figure 8

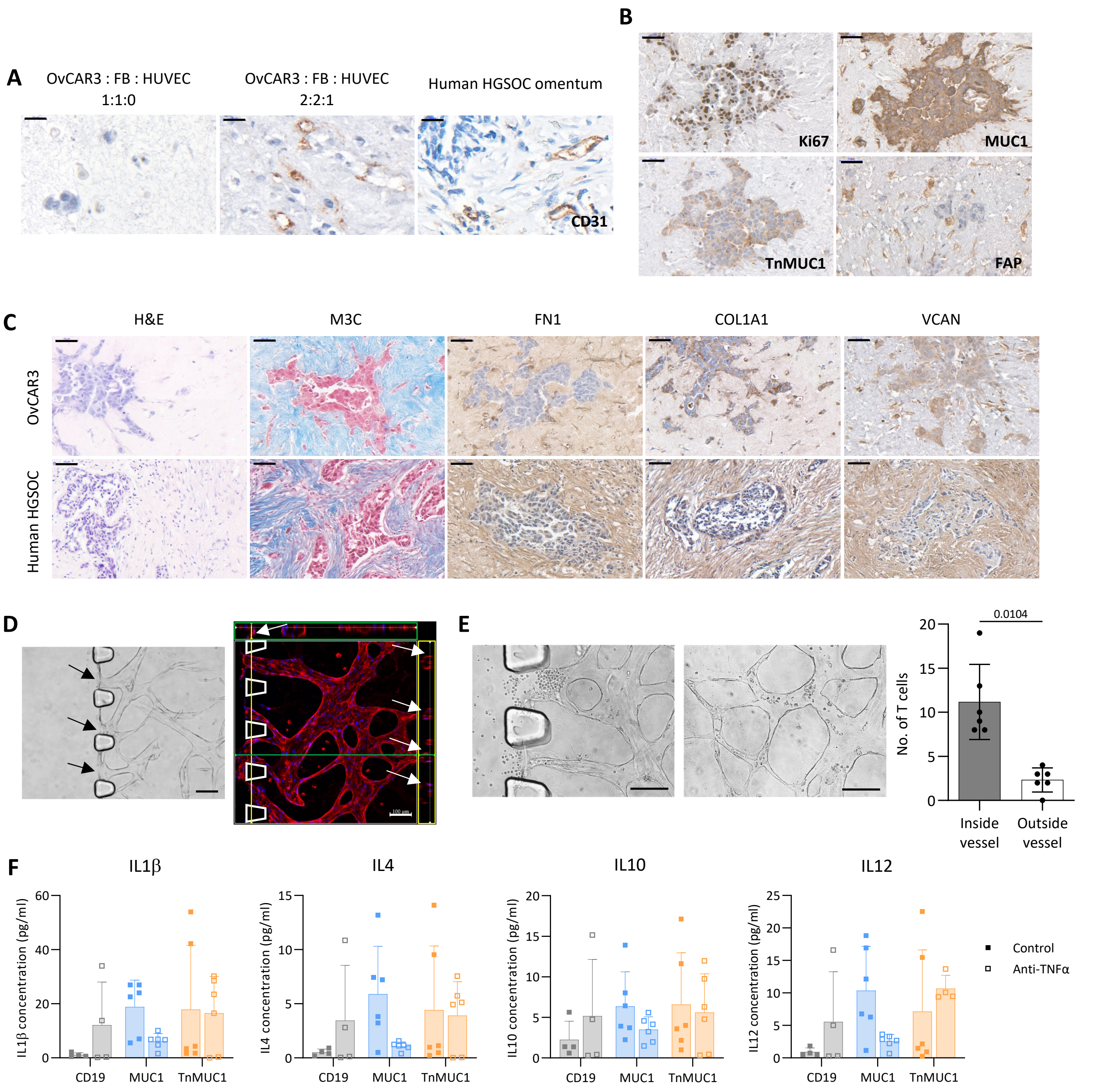

Supplementary Figure 8: Vascularized microfluidic chip to investigate CAR-T cell migration and cytotoxicity. (A) CD31 IHC staining on OvCAR3:FB:HUVEC gels at 1:1:0 and 2:2:1 ratios (n=3) and human HGSOC omental tissues (n=21). Scale bar: 20 µm. **(B)** Expression of Ki67, MUC1, TnMUC1 and FAP in OvCAR3 collagen gels isolated from microfluidic device (n=3). Scale bar: 50 µm. **(C)** Hematoxylin and eosin (H&E), M3C, FN1, COL1A1 and VCAN staining on OvCAR3 gels isolated from the microfluidic device (n=3) and human omental tissues (n=12). Scale bar: 50 µm. **(D)** Phase contrast image (left panel) and cross-sectional immunofluorescence image (right panel) showing lumenised microvasculature (arrows) at the interface of the side and central channels (n=3). Scale bar: 100 µm. Blue = DAPI, Red = CD31. **(E)** Phase contrast images showing T cells within the vessels formed in the microfluidic devices. Scale bar: 100 µm. Data plotted as mean ± SD of two devices per three replicates. Statistics performed using paired t test. **(F)** MSD data showing IL1β, IL4, IL10 and IL12 concentrations in media from microfluidic devices after CAR-T cell and anti-TNFα treatment. Data plotted as mean ± SD for two/three devices per two replicates. Two different CAR-T cell donors were used.
