# Supplementary material for: Human 3D Ovarian Cancer Models Reveal Malignant Cell–Intrinsic and –Extrinsic Factors That Influence CAR T-cell Activity
Source: Cancer Res. 2024 May 31;84(15):2432–49. doi: 10.1158/0008-5472.CAN-23-3007 (PMC11292204; doi:10.1158/0008-5472.CAN-23-3007)
Supplement: Supplementary Table 1 — Fluorophore-conjugated antibodies used for flow cytometry. [file can-23-3007_supplementary_table_1_suppst1.pdf]

Supplementary Table 1: Fluorophore-conjugated antibodies used for flow cytometry.

Antibody	Clone	Dilution	Company	Cat. No.	RRID
CD45 PE-Cy7	H130	1:50	BioLegend	304016	AB_314404
CD3 APC-Cy7	HIT3a	1:200	BioLegend	300317	AB_314053
CD8 BV786	RPA-T8	1:200	BD Bioscience	563823	AB_2687487
CD8 AF700	SK1	1:100	BioLegend	344723	AB_2562789
CD4 BV605	RM4-5	1:300	BioLegend	100547	AB_11125962
CCR2 BV605	K036C2	1:100	BioLegend	357213	AB_2563876
CCR4 BV421	L291H4	1:100	BioLegend	359413	AB_2562435
CCR7 AF647	G043H7	1:50	BioLegend	353217	AB_10913812
CD45RA BV421	HI100	1:50	BioLegend	304129	AB_10965547
CD25 PE-Cy7	BC96	1:150	ThermoFisher	25-0259-41	AB_1257141
CD69 PerCP	FN50	1:200	BioLegend	310927	AB_10696423
HLA-DR BV650	L243	1:200	BioLegend	307650	AB_2563828
PD1 BV711	EH12.2H7	1:100	BioLegend	329928	AB_2562911
PD1 PE-Cy7	EH12.2H7	1:100	BioLegend	329917	AB_2159324
