# Supplementary material for: Human 3D Ovarian Cancer Models Reveal Malignant Cell–Intrinsic and –Extrinsic Factors That Influence CAR T-cell Activity
Source: Cancer Res. 2024 May 31;84(15):2432–49. doi: 10.1158/0008-5472.CAN-23-3007 (PMC11292204; doi:10.1158/0008-5472.CAN-23-3007)
Supplement: Supplementary Table 2 — Primary antibodies used for immunohistochemistry. [file can-23-3007_supplementary_table_2_suppst2.pdf]

Supplementary Table 2: Primary antibodies used for immunohistochemistry.

Antibody	Species	Dilution	Company	Cat. No.	RRID
MUC1 (HMFG2)	Mouse	1:1000	Absolute Antibody	Ab00712-1.1	N/A
TnMUC1 (5E5)	Mouse	1:500	Creative Biolabs	TAB-418MZ	AB_2941837
cIAP1	Goat	1:10	R&D systems	AF8181	AB_2259001
cIAP2	Goat	1:200	R&D systems	AF8171	AB_2243411
CD3	Rabbit	1:800	Dako	A0452	AB_2335677
Cleaved caspase 3	Rabbit	1:100	Cell Signaling	9664S	AB_2070042
Fibronectin	Rabbit	1:500	Sigma-Aldrich	F3648	AB_476976
Versican	Rabbit	1:250	Sigma-Aldrich	HPA004726	AB_1080561
COL1A1	Rabbit	1:150	abcam	ab34710	AB_731684
FAP	Rabbit	1:1000	abcam	ab207178	AB_2864720
αSMA	Mouse	1:2000	Sigma	A5228	AB_262054
Ki67	Mouse	1:50	Dako	M7240	AB_2142367
CD31	Rabbit	1:100	abcam	ab76533	AB_1523298
