# Supplementary material for: Human 3D Ovarian Cancer Models Reveal Malignant Cell–Intrinsic and –Extrinsic Factors That Influence CAR T-cell Activity
Source: Cancer Res. 2024 May 31;84(15):2432–49. doi: 10.1158/0008-5472.CAN-23-3007 (PMC11292204; doi:10.1158/0008-5472.CAN-23-3007)
Supplement: Supplementary Videos legend — Legend for supplementary videos 1-6 [file can-23-3007_supplementary_videos_legend_suppsd.pdf]

Supplementary Video 1: Behavior of CAR-T cells in OvCAR3/FB gels. OvCAR3/FB collagen gels treated with MUC1 CAR-T cells were imaged in real-time for 30 minutes. Representative video from three replicates using three CAR-T cell donors. Red = EpCAM, Blue = Fibronectin, Green = CAR-T cells. Scale bar: 50 µm.

Supplementary Video 2: Behavior of CAR-T cells in G164/FB gels. G164/FB collagen gels treated with MUC1 CAR-T cells were imaged in real-time for 30 minutes. Representative video from three replicates using three CAR-T cell donors. Red = EpCAM, Blue = Fibronectin, Green = CAR-T cells. Scale bar: 50 µm.

Supplementary Video 3: Behavior of CAR-T cells in G164/FB gels without TGFβ-R inhi. G164/FB collagen gels treated with CAR-T cells were imaged in real-time for 30 minutes. Representative video from three replicates using three CAR-T cell donors. Red = EpCAM, Blue = Fibronectin, Green = CAR-T cells. Scale bar: 50 µm.

Supplementary Video 4: Behavior of CAR-T cells in G164/FB gels with TGFβ-R inhi. G164/FB collagen gels treated with TGFβ-R inhi for fourteen days and then with MUC1 CAR-T cells for three further days were imaged in real-time for 30 minutes. Representative video from three replicates using three CAR-T cell donors. Red = EpCAM, Blue = Fibronectin, Green = CAR-T cells. Scale bar: 50 µm.

Supplementary Video 5: Microvasculature formed within fibrin gel penetrated the OvCAR3 collagen gel. z-stack images at the interface between fibrin and collagen gels, showing microvasculature formed within the fibrin gel penetrated OvCAR3 collagen gel (n=3). Red = HUVEC. Scale bar: 100 µm.

Supplementary Video 6: Luminal flow of CAR-T cells through vessels formed in the microfluidic device. Vascularized microfluidic devices treated with MUC1 CAR-T cells were imaged in real-time for two minutes (n=2). Green = CAR-T cells. Scale bar: 20 µm.
